# The Untapped Australasian Diversity of Astaxanthin-Producing Yeasts with Biotechnological Potential—*Phaffia australis* sp. nov. and *Phaffia tasmanica* sp. nov.

**DOI:** 10.3390/microorganisms8111651

**Published:** 2020-10-24

**Authors:** Márcia David-Palma, Diego Libkind, Patrícia H. Brito, Margarida Silva, Nicolás Bellora, Marco A. Coelho, Joseph Heitman, Paula Gonçalves, José Paulo Sampaio

**Affiliations:** 1 UCIBIO, Departamento de Ciências da Vida, Faculdade de Ciências e Tecnologia, Universidade Nova de Lisboa, 2829-516 Caparica, Portugal; marcia.david.palma@duke.edu (M.D.-P.); phbrito@fct.unl.pt (P.H.B.); mri.silva@fct.unl.pt (M.S.); marco.dias.coelho@duke.edu (M.A.C.); pmz@fct.unl.pt (P.G.); 2 Department of Molecular Genetics and Microbiology, Duke University Medical Center, Durham, NC 27710, USA; heitm001@duke.edu; 3 Centro de Referencia en Levaduras y Tecnología Cervecera (CRELTEC), Instituto Andino Patagónico de Tecnologías Biológicas y Geoambientales (IPATEC)—CONICET/Universidad Nacional del Comahue, Bariloche, Rio Negro 8400, Argentina; libkindfd@comahue-conicet.gob.ar (D.L.); nbellora@gmail.com (N.B.)

**Keywords:** *Phaffia*, astaxanthin, phylogenomics, yeast taxonomy, fungal *MAT* genes

## Abstract

*Phaffia* is an orange-colored basidiomycetous yeast genus of the order Cystofilobasidiales that contains a single species, *P. rhodozyma*. This species is the only fungus known to produce the economically relevant carotenoid astaxanthin. Although *Phaffia* was originally found in the Northern hemisphere, its diversity in the southern part of the globe has been shown to be much greater. Here we analyze the genomes of two Australasian lineages that are markedly distinct from *P. rhodozyma*. The two divergent lineages were investigated within a comprehensive phylogenomic study of representatives of the Cystofilobasidiales that supported the recognition of two novel *Phaffia* species, for which we propose the names of *P. australis* sp. nov. and *P. tasmanica* sp. nov. Comparative genomics and other analyses confirmed that the two new species have the typical *Phaffia* hallmark—the six genes necessary for the biosynthesis of astaxanthin could be retrieved from the draft genome sequences, and this carotenoid was detected in culture extracts. In addition, the organization of the mating-type (*MAT*) loci is similar to that of *P. rhodozyma*, with synteny throughout most regions. Moreover, cases of trans-specific polymorphism involving pheromone receptor genes and pheromone precursor proteins in the three *Phaffia* species, together with their shared homothallism, provide additional support for their classification in a single genus.

## 1. Introduction

The basidiomycetous yeast *Phaffia rhodozyma*, formerly known as *Xanthophyllomyces dendrorhous* [1,2], is the only fungus known to produce astaxanthin [3], an economically relevant carotenoid [4]. Initially used solely as an ingredient in aquaculture feeds [5], astaxanthin applications have become diverse in the nutraceutical, cosmetics, food, and feed industries, due to its antioxidant activity together with UV-light protection and anti-inflammatory roles [6]. Although the majority of the commercially available astaxanthin is produced by chemical synthesis, environmental and food safety concerns are driving the development of economically viable natural sources of this carotenoid, involving genetic engineering of *P. rhodozyma* among other approaches [7,8,9]. 

Six genes are known to be involved in the synthesis of astaxanthin in *P. rhodozyma,* which starts in the mevalonate pathway that forms isopentenylpyrophosphate (IPP), the general precursor of all isoprenoids [10]. The transformation of IPP into β-carotene is carried out by four enzymes encoded by genes *IDI*, *CRTE*, *CRTYB* and *CRTI,* with the final conversion of β-carotene into astaxanthin being completed by an astaxanthin synthase (CrtS), which receives the necessary electrons for substrate oxidation from a cytochrome P450 reductase (CrtR) [10]. Although the production of β-carotene in *P. rhodozyma* occurs in a similar way to other carotenogenic fungi, it is the final step performed by CrtS that sets *P. rhodozyma* apart and allows for its unique ability to produce astaxanthin [11]. 

Currently, *P. rhodozyma* is the sole species known in the genus *Phaffia*. This species was originally found in association with exudates of deciduous trees, mostly birch (*Betula*), in Europe, North America, and Japan [1]. For more than three decades it was considered that this yeast existed only in the Northern Hemisphere-birch system, but in 2007 we found *P. rhodozyma* in South America in a new habitat: the stromata of *Cyttaria* spp., an ascomycetous biotrophic parasite of southern beech trees (*Nothofagus* spp.) [12]. Since *Nothofagus* and *Cyttaria* can also be found in Australasia, we investigated the existence of additional populations of *P. rhodozyma* in the Southern Hemisphere, in Queensland and Tasmania (Australia) and in New Zealand’s South Island [13]. This study led to the discovery of an unprecedented diversity of *Phaffia* lineages and indicated that the assortment of genotypes was driven by the type of ecological niche rather than by geography. For example, strains from South American and Australasian *Nothofagus* were found to be genetically related as were the strains from *Betula* from Europe, Asia, and North America [13]. Furthermore, two additional Australasian lineages appeared markedly distinct from *P. rhodozyma.* Here we present a comprehensive genome-based phylogenetic study of *Phaffia* and related genera in the Cystofilobasidiales, and we conclude that these two novel species belong to the *Phaffia* genus and formally describe them as *P. australis* sp. nov. and *P. tasmanica* sp. nov.

## 2. Materials and Methods 

### 2.1. Genome Sequencing and Assembly

Genomic DNA of *Phaffia, Cystofilobasidium*, and *Krasilnikovozyma* (Table 1) was extracted from cultures of single-cell derivatives and paired-end Illumina MiSeq genomic reads were obtained (2 × 300 cycles). For *Cystofilobasidum* spp., an additional short-insert size (~500 bp) library was prepared with a Nextera Kit and subsequently sequenced using the Illumina HiSeq2500 system to generate 151-nt reads. Raw sequence data for the RIKEN genomes (Table 1, Nextera Mate-pair and TrueSeq DNA PCR-free libraries) were downloaded from the European Nucleotide Archive. All raw sequence reads were adapter-trimmed with Trimmomatic v. 0.39 [14]. TrueSeq data was also preprocessed with BBmerge v. 38.73 [15] that cuts adapter sequences and merges overlapping paired reads into single read sequences. Preprocessed reads of *Cystofilobasidium* spp. were assembled with SPAdes v. 3.11.1 [16], with parameter --careful, and k-mer sizes automatically selected based on read length. For *Mrakia aquatica, Trichosporon pullulans, Udeniomyces megalosporus*, and *U. pyricola*, preprocessed reads were assembled with the same software using two different combinations: (i) Nextera mate-pair reads with BBmerge-processed TruSeq reads and (ii) Nextera mate-pair reads with Trimmomatic-processed TruSeq reads. Resulting assemblies for each genome were assessed for quality with QUAST v. 5.0. [17], and the assemblies with the largest genome size and N50 value were retained. Preprocessed sequence reads of *Tausonia pamirica* were assembled into scaffolds using ABYSS v. 2.0.2 [18] with default parameters. ABYSS was run with multiple k (kmer) parameter values and the optimal value of k was assessed by inspecting the assembly contiguity statistics. Small contigs of less than 1 kb were discarded from all final assemblies. *Ab initio* prediction of protein-coding genes and annotation of all final assemblies were performed with AUGUSTUS v. 3.3.3 [19] using the default gene structure training set for *Cryptococcus deneoformans*.

### 2.2. Orthology Mapping, Genomic Diversity, and Phylogenetic Analyses

Orthology mapping was carried out using all-against-all BLASTP (NCBI Blast-2.2) searches and a Markov cluster algorithm (OrthoMCL v. 1.4; [20]) with an inflation factor (F) of 1.5, and a minimum pairwise sequence alignment coverage of 50% as implemented in the Get_Homologues package [21]. From this analysis, we retained all clusters that contained one single orthologous copy in all analyzed genomes to form a dataset for the phylogenetic analysis of single-copy core proteins. Protein sequences of each cluster were aligned with MAFFT v. 7.407 [22] using the G-INS-I method and default parameter values, trimmed with BMGE v. 1.12 [23] using the amino acid option, and finally concatenated into a single dataset. Alignment-free pairwise distances, Kr, that are based on the rate of substitutions between two unaligned sequences using the average shortest unique substring (shustring) [24] were calculated with GenomeTools [25]. For these calculations, a restrictive threshold of 0.3 was applied to discard inconsistent values between genome triads at high Kr levels [26]. The phylogenetic analysis was carried out with IQ-TREE v. 1.6.12 [27] using maximum-likelihood inference. Model choice was determined by ModelFinder [28] and branch support was estimated using fast bootstrap approximation with NNI optimization [29], both implemented in IQ-TREE. Phylogenies of the pheromone receptors and pheromone precursor proteins were inferred by NJ using the JTT matrix-based method as implemented in MEGA 5.1 [30] using 1000 bootstrap replicates. The corresponding proteins of *C. deneoformans* were used as outgroups for each of the phylogenies: AAN75624 (Ste3a), XP_570116 (Ste3α), AAG42766 (MFa1) and XP_570122 (MFα1).

### 2.3. Search for Relevant Genes

The search for genes pertaining to the astaxanthin synthesis pathway in the draft genomes of *P. australis* and *P. tasmanica* was performed by TBLASTN using the correspondent proteins from *P. rhodozyma* as the query. The same approach was used to search for mating type (*MAT*) genes in the new species. Putative orthologs were named according to the protein accession number of *P. rhodozyma* CBS 6938. Synteny conservation across species was assessed manually based on the predicted annotations and confirmed by high-scoring BLASTP hits in GenBank. The transmembrane regions in the pheromone receptor (*PR*) proteins were predicted by HMMTOP software [31]. For the deduced Hd1 and Hd2 proteins, homeodomain regions were determined using the InterPro server [32], while nuclear localization signals (NLS) were predicted using the SeqNLS server [33] with a 0.5 cutoff. Potential alpha-helices were predicted by Jpred4 [34], and searches for coiled-coil dimerization motifs were conducted using COILS [35] with a sliding window of 28, weighing option and probability ≥ 90% [36].

Predicted pheromone precursor proteins sequences from the three *Phaffia* species were aligned with ClustalW as implemented in BioEdit [37] in order to predict the cleavage site giving rise to the peptide moiety of the mature pheromone.

### 2.4. Phenotypic Characterization

The characterization of vegetative and sexual structures was carried out in YPD (yeast extract 1% *w/v*, peptone 2% *w/v*, glucose 2% *w/v*), and on YM agar (yeast extract 0.3% *w/v*, malt extract 0.3% *w/v*, peptone 0.3% *w/v*, glucose 1% *w/v*, agar 2% *w/v*), corn meal agar (Difco), and ribitol agar (ribitol 0.5% *w/v* and agar 2.5% *w/v*). Microscopic observations were made using a Leica DMR microscope equipped with differential interference contrast optics. Dissection of basidiospores was performed using a Zeiss Scope A1 Micromanipulator. Physiological and biochemical characteristics were examined according to standard protocols [38] and were performed in triplicate. The extraction of carotenoids was performed as previously reported [39], and the presence of astaxanthin was assessed by HPLC-PAD using standard techniques [40]. 

## 3. Results

### 3.1. The Divergent Australasian Lineages Belong to the Genus Phaffia

**Figure 1:**
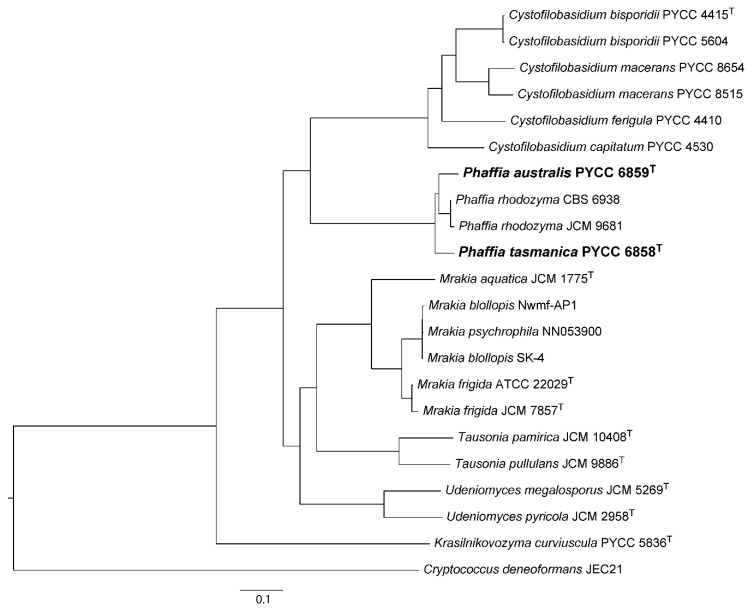
Genome-based phylogenetic placement of *Phaffia australis* and *P. tasmanica* within the Cystofilobasidiales. The phylogeny was inferred from a concatenated alignment of 485 amino acid sequences corresponding to single copy core genes from all taxa (including *Cryptococcus deneoformans* employed to root the tree), using the LG+F+I+G4 model of sequence evolution and the maximum likelihood method as implemented in IQ-TREE. The analysis includes representative species of six genera of the Cystofilobasidiales (*C. cystofilobasidium, K. krasilnikovozyma, M. mrakia, P. phaffia, T. tausonia,* and *U. udeniomyces*) with the two new *Phaffia* species depicted in bold. All branches have 100% bootstrap support (fast bootstrap with NNI optimization), and branch lengths correspond to the expected number of substitutions per site.

To assess the phylogenetic placement of the new lineages at the genus and species level, we used a concatenated alignment of the amino acid sequences of 485 single copy core genes. These sequences were retrieved from the draft genome sequences obtained in this study and from public databases (Table 1). Representatives of six genera of the Cystofilobasidiales were included in the phylogeny depicted in Figure 1 that confirmed that *Cystofilobasidium* and *Phaffia* are sister genera. The phylogeny also provided a robust topology for the remaining lineages within the order Cystofilobasidiales, that improves previous analyses of this group [41]. The clear demarcation at the genus level shown in Figure 1 suggests that the two new Australasian species are adequately described in the genus *Phaffia*, and also confirms that they represent new species. With respect to the level of nucleotide substitutions among multiple strains of *P. australis, P. tasmanica* and *P. rhodozyma* in the complete ITS sequence, a recognized DNA barcode for fungi [42,43], we measured 25–36 nucleotide substitutions in pairwise comparisons involving the three species. For example, in the sister genus *Cystofilobasidium,* the number of nucleotide substitutions recorded between the different species currently recognized in this genus ranged between 11 and 26, representing an equivalent level of divergence. Our previous results obtained using both housekeeping genes and genes encoding enzymes of the astaxanthin biosynthetic pathway also support the recognition of two new *Phaffia* species, as the divergence between the proposed three species clearly exceeds the documented divergence between the various populations of *P. rhodozyma* [13]. 

**Figure 2:**
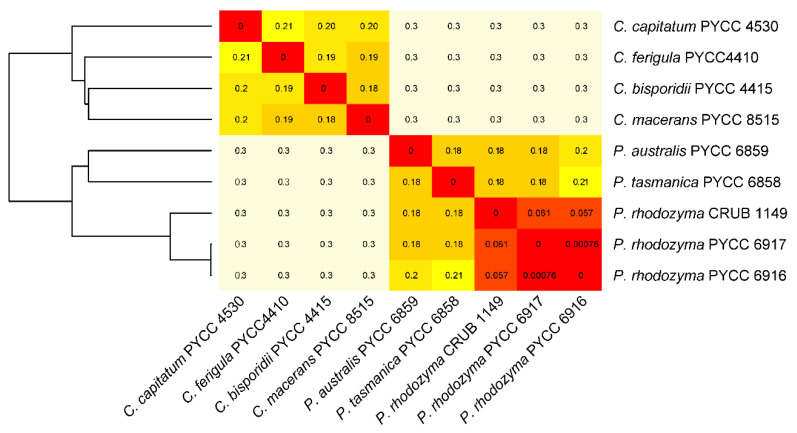
Pairwise divergence using the Kr alignment-free method between the three *Phaffia* (*P*.) species and comparison with four species of *Cystofilobasidium* (*C*.). Heat-map of divergence values (red, lowest divergence; pale yellow, highest divergence). The dendrogram on the left was based on Euclidean distances and average clustering.

Draft genome sequences of representatives of *Cystofilobasidium* and *Phaffia* were also used to estimate alignment-free pairwise distances (Figure 2). Low divergence values (Kr) of less than 0.07 were obtained for comparisons involving three genomes of *P. rhodozyma*, whereas congeneric genome-wide divergence ranged between 0.18 and 0.21, both in *Phaffia* and in *Cystofilobasidium*. 

Intergeneric divergence was always higher (0.3) than congeneric divergence. These values are in accordance with measurements made with the same approach in ascomycetous and basidiomycetous yeasts [44]. A sliding window analysis of the longest scaffold revealed relatively constant levels of divergence between *P. rhodozyma* and the two novel species (Figure S1), and a similar level of pairwise divergence between the *Phaffia* genomes was found using an alignment-free method (Figure S1). Taken together, these analyses support the view that the two Australasian lineages are genetically divergent from each other and from *P. rhodozyma*, and they show levels of interspecies genetic divergence similar to those observed among species of the sister genus *Cystofilobasidium* (Figure 2 and Figure S1).

### 3.2. Phenotypic Characterisation of P. australis sp. nov and P. tasmanica sp. nov.

**Figure 3:**
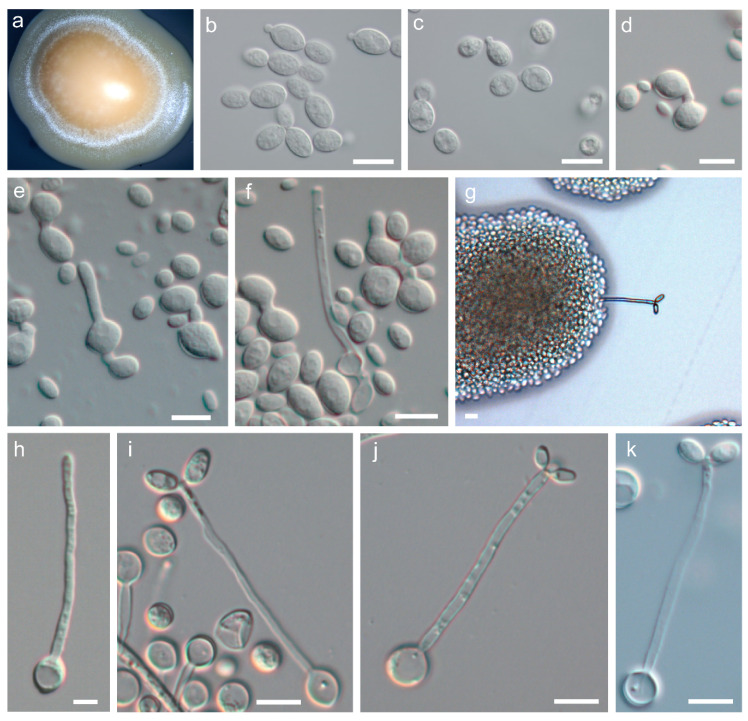
Salient micromorphological features of *Phaffia australis* sp. nov. and *Phaffia tasmanica* sp. nov. Orange-salmon colored colony of *P. tasmanica* PYCC 6947 with a whitish velvet-like margin of aerial basidia and basidiospores (**a**). Yeast cells of *P. australis* PYCC 6859^T^ (**b**) and *P. tasmanica* PYCC 6858^T^ (**c**) on YPD after 3 days at 20 °C. Cell-cell conjugation (**d**) and subsequent basidium formation (**e**–**g**) of *P. australis* PYCC 6859^T^. Early stage of basidium development from a single cell of *P. tasmanica* PYCC 6948 (**h**) and mature basidia with basidiospores (**i**–**k**) of PYCC 6858^T^. All stages of sexual development were studied on ribitol agar incubated at 18 °C and observations were conducted after 3–10 days. Scale bars correspond to 10 µm.

Vegetative growth in both species occurred predominantly by budding (Figure 3b–c). True hyphae were not formed although pseudohyphae were produced on corn meal agar and ribitol agar. With respect to sexual reproduction, both *P. australis* and *P. tasmanica* formed basidia that exhibited many of the distinctive features previously reported for *P. rhodozyma* [2], namely a slender and filiform structure (Figure 3g,i–k) that originates directly from yeast cells (Figure 3f,h), grows aerially from the yeast colony (Figure 3a) and has terminal basidiospores (Figure 3g,i–k); hyphae were completely absent. However, some details of the reproductive cycle are unique to each of the different species. For instance, in *P. rhodozyma,* pedogamy (conjugation between the parent cell and its bud) precedes the formation of the basidium [2], whereas in *P. australis*, conjugation between two independent cells is the norm (Figure 3d–e), and in *P. tasmanica* most basidia arise from a single cell, with no apparent conjugation (Figure 3h). Pedogamy was also observed in both novel species but only rarely. Like in *P. rhodozyma*, the sexual stage of the two novel species is triggered by the presence of ribitol as a sole carbon source in the culture medium. Basidia produced by these two novel species are non-septate, slender, and form two to four basidiospores terminally on minute pegs (Figure 3i–k). By micromanipulation of basidiospores of *P. australis* and *P. tasmanica*, we observed that cultures derived from single basidiospores were able to complete the sexual life cycle, thus confirming that the new species are homothallic, like *P. rhodozyma*. 

The standard physiological and biochemical characterization of the two novel species is shown in Table 2. The two new *Phaffia* species were able to ferment glucose and sucrose, an uncommon characteristic for basidiomycetous yeasts but a trait also present in *P. rhodozyma*. Additionally, *P. tasmanica* fermented raffinose, unlike the other two species. *Phaffia australis* differed from *P. tasmanica* and *P. rhodozyma* in its ability to grow on xylitol and ethylamine (Table 2). The inability to grow on D-xylose and D-glucitol were unique to *P. tasmanica*. Similarly to *P. rhodozyma,* the new species were able to grow at 25 °C but were unable to grow at 30 °C. Therefore, the phenotypic hallmarks of the genus *Phaffia* are the unique formation of basidia directly from yeast cells, a homothallic life cycle, and the ability to ferment glucose and other simple sugars. 

### 3.3. Astaxanthin Production

Cultures of representative strains of the three species have a similar orange to salmon color that can vary in intensity, depending on the age of the culture and exposure to light, i.e., older cultures or those more exposed to light tend to become more pigmented. Previously, *P. rhodozyma* was the only yeast species known to produce astaxanthin. To ascertain whether this characteristic was also present in the new species, the ability of *P. australis* and *P. tasmanica* to produce this carotenoid was evaluated. Sequences of the six genes of *P. rhodozyma* involved in the biosynthetic pathway of astaxanthin (*IDI, CRTI, CRTYB, CRTE, CRTR* and *CRTS*) were used to query the genomes of the type strains of the new species. We employed TBLASTN and used the correspondent proteins from *P. rhodozyma* as queries (CAA75796, AAY33922, AAY33923, CAA75240, ABA43719, ACI43097). All six genes were readily retrieved from the two draft genome sequences. The genes appeared to encode functional enzymes, exhibiting 90–98% amino acid sequence identity to those of *P. rhodozyma* (Figure S2). Although genes encoding secondary metabolite biosynthetic pathways are often clustered in fungi [45], the astaxanthin genes do not appear to be clustered in the genomes of the two new species and are instead scattered across different contigs (Table S1). This same pattern of gene organization has also been observed in *P. rhodozyma* [26]. The presence of astaxanthin in culture extracts of *P. australis* and *P. tasmanica* was confirmed by HPLC-PAD using *P. rhodozyma* CBS 7918 as a positive control.

### 3.4. Organization of MAT Loci

**Figure 4:**
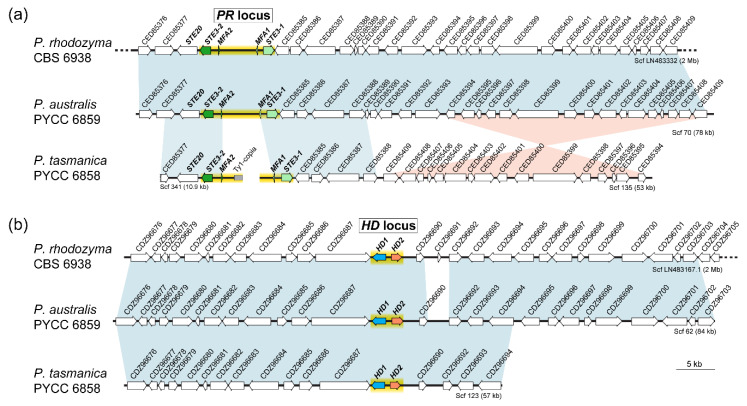
Organization of the mating-type (*MAT)* loci in the three *Phaffia* species. Scaffolds encompassing *PR* (**a**) and *HD* (**b**) genes in the different *Phaffia* species are represented. Genes are depicted as arrows indicating the direction of transcription and are identified by the protein accession number of their putative orthologs in *P. rhodozyma* CBS 6938. Mating-type genes, including pheromone receptor genes (*STE3*), pheromone precursor genes (*MFA*), and homeodomain transcription factor genes (*HD1* and *HD2*) are color-coded, while other genes are depicted in white. The regions spanning the proposed *HD* and *PR* loci are highlighted in yellow. In *P. tasmanica*, the *PR* locus is located at the end of two scaffolds, which are possibly interrupted by transposable elements. Scaffolds ending with a dotted line indicate that the scaffold is only partially represented. Blocks of synteny with orthologs in the same or inverted orientation are depicted by blue or pink bars, respectively.

The same approach as above was used to search for mating type (*MAT*) genes in the new species, employing the *P. rhodozyma* query proteins CED85384 (*STE3-1*), CED85379 (*STE3-2*), CDZ96688 (*HD1*), and CDZ96689 (*HD2*). We detected *MAT* genes similar to those present in *P. rhodozyma* in the draft genomes of *P. australis* and *P. tasmanica*. Predicted amino acid sequences of the pheromone receptors, Ste3-1 and Ste3-2, revealed the characteristic seven transmembrane domains in both species, with the exception of Ste3-2 of *P. australis* for which eight transmembrane domains were predicted (Figure S3). The predicted amino acid sequences of the homeodomain transcription factors, Hd1 and Hd2 of *P. australis*, present features similar to the Hd1/Hd2 pair of *P. rhodozyma*, with a nuclear localization signal (NLS) present only in the Hd2 protein and coiled-coils present only in the C-terminal region of Hd1. For *P. tasmanica,* NLS were predicted in both proteins, while coiled-coils were only present in the C-terminal region of Hd1, as in the other *Phaffia* species [46] (Figure S4). The organization of the *MAT* loci of *P. australis* and *P. tasmanica* is similar to that of *P. rhodozyma*, with synteny maintained throughout the scaffolds containing the *MAT* regions, except for a large inversion that took place in *P. tasmanica* close to the *PR* locus (Figure 4). 

**Figure 5:**
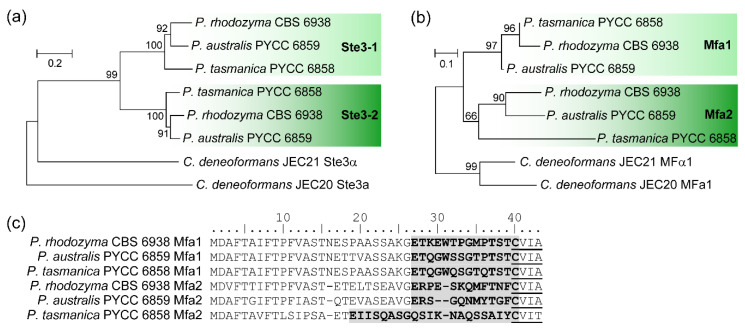
Trans-specific polymorphism of pheromone receptor genes in the genus *Phaffia*. Phylogeny of pheromone receptors (**a**) and pheromone precursor proteins (**b**) from all *Phaffia* species, and alignment of pheromone precursor proteins (**c**). Predicted mature pheromones are highlighted in grey with bold font and the C-terminal motif for posttranslational processing is underlined.

Moreover, a phylogenetic analysis of pheromone receptors and pheromone precursor proteins from the three *Phaffia* species revealed trans-specific polymorphisms (Figure 5). This means that the *STE3-1* (or *MFA1*) alleles of the different *Phaffia* species are more similar to each other than to the alternate *STE3-2* (or *MFA2*) alleles present in the genome of each *Phaffia* species. Pheromone receptor alleles have been documented in various lineages of Basidiomycota [47] and they are usually deeply trans-specific, with alleles from distantly related species or even members of distinct Classes grouping in the same allelic clade [48]. Interestingly, the trans-specific polymorphism of *Phaffia STE3* alleles appears to not be as deep as in other basidiomycete lineages (Figure 5) because both alleles are still more closely related to each other than to alternate alleles of the more distantly related species *C. deneoformans*. Therefore, we suggest the trans-specific polymorphism observed in *Phaffia* is comparatively recent and was initiated before the radiation of the *Phaffia* species but after the divergence of the *Phaffia* ancestor from the other Cystofilobasidiales lineages. This suggests that the two pheromone/receptor alleles in *Phaffia* may have a different evolutionary origin as compared to other basidiomycete pheromone/receptors [46]. Finally, comparative analysis of the pheromone precursor proteins of the three *Phaffia* species showed Mfa1 to be more conserved than Mfa2, especially Mfa2 of *P. tasmanica* (Figure 5c).

### 3.5. Taxonomy 

#### 3.5.1. Description of *Phaffia australis* sp. nov. M. David-Palma, D. Libkind, P. Gonçalves and J.P. Sampaio 

Mycobank accession: MB 836706. 

Whole genome data: PRJNA371751.

Genbank LSU and ITS sequences: KR108929 and JN637116, respectively.

Etymology: australis (Lat.), meaning southern and referring to Australia and New Zealand, the countries where this species was originally found.

After 3 days at 20 °C on YMA, cultures have an orange to salmon color and a butyrous texture. After 3 days at 20 °C on YPD, cells are ellipsoidal (4–8 × 8–16 μm) and proliferation is done by budding at the distal ends of the cell (Figure 3b). On corn meal agar or ribitol agar after two weeks at 18 °C, pseudohyphae can be present but true hyphae are not produced. Basidia can be observed on ribitol agar after incubation at 18 °C for four days. Cell-cell conjugation initiates the development of the sexual stage (Figure 3d). Basidia originate directly from one of the conjugated cells (Figure 3e–f) and are slender (2–3 × 50–80 μm) with a slight swelling at the apex. Ellipsoidal basidiospores (5–6 × 10–11 μm) range from two to six, are formed terminally in the basidium (Figure 3g) and germinate by budding. The physiological and biochemical profile is shown in Table 2, and the phylogenetic placement based on whole-genome data is shown is Figure 1. 

The holotype (PYCC 6859^H^) is permanently maintained in a metabolically inactive state in the Portuguese Yeast Culture Collection, Caparica, Portugal; the type strain was deposited in the same collection (PYCC 6859^T^) and in the collection of the Westerdijk Fungal Biodiversity Institute (CBS 14095^T^), Utrecht, the Netherlands. The strain ZP 938 (PYCC 6859^T^) was isolated in November 2009 from leaves of *Nothofagus mooreii* collected in the Lamington National Park, Queensland, Australia. 

#### 3.5.2. Description of *Phaffia tasmanica* sp. nov. M. David-Palma, D. Libkind, P. Gonçalves and J.P. Sampaio 

Mycobank accession: MB 836707. 

Whole genome data: PRJNA371754.

Genbank LSU and ITS sequences: KT223097 and JN637120, respectively.

Etymology: tasmanica (Lat.), pertaining to Tasmania, the island where this species was originally found.

After 3 days at 20 °C on YMA, cultures have an orange to salmon color and a butyrous texture (Figure 3a). After 3 days at 20 °C on YPD, cells are ellipsoidal (4–6 × 7–9 μm) and proliferation is done by budding at the distal ends of the cell (Figure 3c). On corn meal agar or ribitol agar after two weeks at 18 °C, pseudohyphae can be present but true hyphae are not produced. Basidia form on ribitol agar after incubation at 18 °C for four days. Typically, basidia develop from single cells (Figure 3 h) and cell-cell conjugation does not occur. Basidia are slender (2–3 × 50–90 μm) with a slight swelling at the apex (Figure 3 i–k). The basidiospores are subglobose to ellipsoidal (2–3 × 5–8 μm), range from two to six, are formed terminally in the basidium (Figure 3g), and germinate by budding. The physiological and biochemical profile is shown in Table 2, and the phylogenetic placement based on whole-genome data is shown is Figure 1. 

The holotype (PYCC 6858^H^) is permanently maintained in a metabolically inactive state in the Portuguese Yeast Culture Collection, Caparica, Portugal; the type strain was deposited in the same collection (PYCC 6858^T^) and in the collection of the Westerdijk Fungal Biodiversity Institute (CBS 14096^T^), Utrecht, the Netherlands. The strain ZP 875 (PYCC 6858^T^) was isolated in November 2009 from fruiting bodies of *Cyttaria gunnii* on *Nothofagus cunninghamii* in Mount Field National Park in Tasmania (Australia). 

## 4. Concluding Remarks

The genus *Phaffia* represents a unique lineage of basidiomycetous yeasts that form basidia directly from yeast cells, lack hyphae throughout the life cycle, and produce astaxanthin, a carotenoid of biotechnological interest. Here, we expand the mapped diversity of the genus by adding to the single species know so far, *P. rhodozyma*, two novel taxa, *P. australis* and *P. tasmanica*. As a consequence, the phylogeography and the ecological range of the genus *Phaffia* is considerably expanded. *Phaffia rhodozyma* was originally discovered in the slimy exudates of *Betula* spp. and a few other deciduous trees in the Northern Hemisphere. This restricted view of *Phaffia* distribution and ecology started to change in 2007 when *P. rhodozyma* was found in temperate forests in South America, associated with the *Cyttaria-Nothofagus* system [12]. A subsequent investigation of *Nothofagus* forests in Australasia revealed not only the presence of *P. rhodozyma,* but also divergent lineages whose taxonomic position is clarified here [13]. Therefore, while the three species currently known in the genus *Phaffia* are present in the Southern hemisphere and associated with the *Cyttaria*- *Nothofagus* system, only one of these species, *P. rhodozyma,* is also found in the Northern hemisphere. Given that Australasia has the highest diversity and the greatest level of endemism, we suggested that the genus evolved originally in this region [13]. Moreover, the association with *Cyttaria*–*Nothofagus* seems to be an ancestral trait in the genus because all three known species were isolated from this niche. The data presently available suggest that while *P. australis* and *P. tasmanica* are endemic to Australasia, *P. rhodozyma* is cosmopolitan and present in Australasia, South America, and in the Holarctic region. 

Based on the overall similarity of the *PR* and *HD* regions and the *MAT* genes of *P. tasmanica* and *P. autralis* to those of *P. rhodozyma*, and the fact that these two new species also reproduce through a homothallic sexual cycle, it seems reasonable to propose a common genetic basis for the homothallic behavior of the three species, which was likely present in their most recent common ancestor.

Our phylogenetic analysis included two genomes of *P. rhodozyma*. One of them (PYCC 6917 = JCM 9681 = CBS 7918) corresponds to the type strain of *Xanthophyllomyces dendrorhous*, the nomenclatural designation of the sexual stage of *Phaffia rhodozyma*, which was originally described as asexual [2]. Since the abolishment of the dual nomenclature system for fungi [49], the designations of asexual and sexual morphs have been merged into a single one, and *P. rhodozyma* was retained over *X. dendrorhous* [41]. However, given that the type strain of *P. rhodozyma* (PYCC 6914 = CBS 5905) has mixed ancestries at the population level, which was revealed by an enriched number of heterozygous sites in multi-locus sequence typing studies [12,13], this strain may be inappropriate for use as a reference genome of the species. In contrast, PYCC 6917 shows no mixed ancestry and is therefore much more suitable to be regarded as the reference genome of *P. rhodozyma*. Because it is not possible to change the type (strain) of a species according to the nomenclature rules, we suggest that PYCC 6917 should be informally regarded as the reference genome (and strain) of *Phaffia rhodozyma*.

## Figures and Tables

**Table 1:** List of draft genomes and correspondent cultures used in this work.

Species	Sequenced Strain	Other Collections	Genome Data (BioProject)	Origin
*Cryptococcus deneoformans*	JEC21		PRJNA13856	NCBI
*Cystofilobasidium bisporidii*	PYCC 5604	CBS 6347	PRJNA371778	This study
*Cystofilobasidium bisporidii*	PYCC 4415^T^	CBS 6346^T^	PRJNA371780	This study
*Cystofilobasidium capitatum*	PYCC 4530	CBS 7420	PRJNA371774	This study
*Cystofilobasidium ferigula*	PYCC 4410	CBS 7201	PRJNA371786	This study
*Cystofilobasidium macerans*	PYCC 8515	CBS 6532	PRJNA371809	This study
*Cystofilobasidium macerans*	PYCC 8654	CBS 2425	PRJNA371814	This study
*Krasilnikovozyma curviuscula*	PYCC 5836^T^		PRJNA371818	This study
*Mrakia aquatica*	JCM 1775^T^		PRJDB3647	RIKEN BioResource Center
*Mrakia blollopis*	SK-4		PRJDB3253	NCBI
*Mrakia blollopis*	Nwmf-AP1		RRJNA268263	NCBI
*Mrakia frigida*	JCM 7857^T^		PRJDB3713	RIKEN BioResource Center
*Mrakia frigida*	ATCC 22029^T^		PRJNA334195	JGI
*Mrakia psychrophila*	NN053900		PRJNA304674	NCBI
*Phaffia australis*	PYCC 6859^T^	CBS 14095^T^	PRJNA371751	This study
*Phaffia rhodozyma*	CBS 6938	PYCC 6916	PRJEB6925	NCBI
*Phaffia rhodozyma*	JCM 9681	PYCC 6917	PRJDB3716	RIKEN BioResource Center
*Phaffia tasmanica*	PYCC 6858^T^	CBS 14096^T^	PRJNA371754	This study
*Tausonia pamirica*	JCM 10408^T^		PRJDB3689	RIKEN BioResource Center
*Tausonia pullulans*	JCM 9886^T^		PRJDB3678	RIKEN BioResource Center
*Udeniomyces megalosporus*	JCM 5269^T^		PRJDB3720	RIKEN BioResource Center
*Udeniomyces pyricola*	JCM 2958^T^		PRJDB3672	RIKEN BioResource Center

Acronyms of culture collections: ATCC, American Type Culture Collection, USA; CBS, Westerdijk Fungal Biodiversity Institute (Centraalbureau voor Schimmelcultures), Utrecht, the Netherlands; JCM, Japan Collection of Microorganisms, Riken Bioresource Research Center, Japan; PYCC, Portuguese Yeast Culture Collection, Caparica, Portugal.

**Table 2:** Physiological characteristics of *P. australis* sp. nov. PYCC 6859^T^, PYCC 6943 and PYCC 6944, *P. tasmanica* sp. nov. PYCC6858^T^, PYCC 6947 and PYCC 6948, and comparison with *P. rhodozyma* CBS 7918^T^ and CBS 5905 (+, positive; -, negative; D, delayed; V, variable; W, weak results).

Fermentation	*P. australis*	*P. tasmanica*	*P. rhodozyma*
D-Glucose	+	+	+
D-Galactose	-	-	-
D-Xylose	-	-	-
Sucrose	+, W	+	D, -
Maltose	-	-	D, -
α,α-Trehalose	-	-	D, -
Melibiose	-	-	-
Lactose	-	-	-
Methyl-α-D-glucoside	-	-	-
Cellobiose	-	-	D, -
Melezitose	-	+	D, -
Raffinose	-	W	-
Inulin	-	-	-
Soluble Starch	-	-	-
Assimilation of carbon compounds			
D-Glucose	+	+	+
D-Galactose	-	D, W	-
L-Sorbose	-	-	D, -
D-Glucosamine	-	-	-
D-Ribose	-	-	D, -
D-Xylose	+	-	+
L-Arabinose	+	+	+
D-Arabinose	-	-	D, -
L-Rhamnose	-	-	D, -
Sucrose	+	+	+
Maltose	+	+	+
α,α-Trehalose	+	+	+
Methyl-α-D-glucoside	+	+	D, -
Cellobiose	+	+	+
Salicin	+	+	+
Melibiose	+	+	-
Lactose	-	-	-
Raffinose	+	+	+
Melezitose	+	+	+
Inulin	-	-	-
Soluble Starch	+	+	+
Glycerol	+	+	+
Erythritol	-	-	-
Ribitol	D	-	D, -
Xylitol	+	-	-
D-Glucitol	+	-	D
D-Mannitol	+	+	+
Galactitol	-	-	-
Inositol	-	-	-
Glucono-δ-lactone	+	+	+
D-Gluconic acid	+	+	+
D-Glucuronic acid	+	w	+, D
D,L-Lactic acid	-	-	V
Succinic acid	+	+	+
Citric acid	+	+	+
D-Tartaric acid	-	-	-
*m*-Tartaric acid	-	-	-
Saccharic acid	-	-	-
Mucic acid	-	-	-
Methanol	-	-	-
Ethanol	+	+	+
Assimilation of nitrogen compounds			
Nitrate	-	-	-
Nitrite	-	-	-
Ethylamine	+	-	-
L-Lysine	+	+	+
Cadaverine	W	W	+
Creatine	-	-	-
Creatinine	-	-	-
			
Other tests			
Growth in vitamin-free medium	-	-	-
Growth in the presence of 0.01% cycloheximide	-	-	-
Growth in the presence of 0.1% cycloheximide	-	-	-
Growth at 25 °C	+	+	+
Growth at 30 °C	-	-	-
Formation of starch-like compounds	+	+	+
Hydrolysis of urea	+	+	+
Colour reaction with Diazonium Blue B	+	+	+

## Supplementary Materials

The following are available online at https://www.mdpi.com/2076-2607/8/11/1651/s1, Table S1: Localization of the genes encoding enzymes of the astaxanthin biosynthetic pathway in the draft genomes of *P. australis* and *P. tasmanica*, Figure S1: (A) Sequence similarity between the new species *Phaffia australis* and *P. tasmanica* and *P. rhodozyma* along the longest scaffold (scaffold 52) of *P. rhodozyma* CBS 6938. The left scale represents the divergence measured by a sliding window approach to *P. rhodozyma* CBS 7918 (red), *P. rhodozyma* CRUB 1149 (blue), *P. australis* PYCC 6859 (yellow) and *P. tasmanica* PYCC 6858 (magenta). The mean divergence value for each strain is shown using the color-codes indicated above. The right Y scale represents the percentage of Ns in the reference sequence, which are indicated as grey bars (1.5% of the scaffold sequence corresponded to Ns). (B) Genomic sequence divergence using an alignment-free method. Heat map and clustering with pairwise distances (Kr values) between the draft genomes of *P. rhodozyma* CBS 7918, CRUB 1149 and CBS 6938, *P. tasmanica* PYCC 6858, *P. australis* PYCC 6859, *Cystofilobasidium bisporidii* PYCC 4415, *Cy. macerans* PYCC 8515, *Cy. capitatum* PYCC 4530 and *Cy. ferigula* PYCC 4410, Figure S2: Amino acid alignments of the proteins involved in the astaxanthin biosynthetic route in *Phaffia* spp., Figure S3: Predicted proteins of the pheromone receptor genes of *P. australis* and *P. tasmanica*. The transmembrane domains of each sequence are highlighted in blue, Figure S4: Predicted proteins of the homeodomain transcription factor genes of *P. australis* and *P. tasmanica*. The secondary organization is highlighted.

## References

[1] Miller M.W., Yoneyama M., Soneda M. (1976). *Phaffia*, a new yeast genus in the *Deuteromycotina* (*Blastomycetes*). Int. J. Syst. Evol. Microbiol..

[2] Golubev W.I. (1995). Perfect state of *Rhodomyces dendrorhous* (*Phaffia rhodozyma*). Yeast.

[3] Alcaíno J., Baeza M., Cifuentes V., Stange C. (2016). Carotenoid distribution in nature. Carotenoids in Nature: Biosynthesis, Regulation and Function.

[4] Schmidt I., Schewe H., Gassel S., Jin C., Buckingham J., Humbelin M., Sandmann G., Schrader J. (2011). Biotechnological production of astaxanthin with *Phaffia rhodozyma*/*Xanthophyllomyces dendrorhous*. Appl. Microbiol. Biotechnol..

[5] Foss P., Storebakken T., Schiedt K., Liaaen-Jensen S., Austreng E., Streiff K. (1984). Carotenoids in diets for salmonids. Aquaculture.

[6] Guerin M., Huntley M.E., Olaizola M. (2003). *Haematococcus* astaxanthin: Applications for human health and nutrition. Trends Biotechnol..

[7] Loto I., Gutierrez M.S., Barahona S., Sepulveda D., Martinez-Moya P., Baeza M., Cifuentes V., Alcaino J. (2012). Enhancement of carotenoid production by disrupting the C22-sterol desaturase gene (*CYP61*) in *Xanthophyllomyces dendrorhous*. BMC Microbiol..

[8] Gassel S., Breitenbach J., Sandmann G. (2014). Genetic engineering of the complete carotenoid pathway towards enhanced astaxanthin formation in *Xanthophyllomyces dendrorhous* starting from a high-yield mutant. Appl. Microbiol. Biotechnol..

[9] Ledetzky N., Osawa A., Iki K., Pollmann H., Gassel S., Breitenbach J., Shindo K., Sandmann G. (2014). Multiple transformation with the *crtYB* gene of the limiting enzyme increased carotenoid synthesis and generated novel derivatives in *Xanthophyllomyces dendrorhous*. Arch. Biochem. Biophys..

[10] Barredo J.L., García-Estrada C., Kosalkova K., Barreiro C. (2017). Biosynthesis of astaxanthin as a main carotenoid in the heterobasidiomycetous yeast *Xanthophyllomyces dendrorhous*. J. Fungi.

[11] Rodríguez-Sáiz M., De La Fuente J.L., Barredo J.L. (2010). *Xanthophyllomyces dendrorhous* for the industrial production of astaxanthin. Appl. Microbiol. Biotechnol..

[12] Libkind D., Ruffini A., Van Broock M., Alves L., Sampaio J.P. (2007). Biogeography, host specificity, and molecular phylogeny of the basidiomycetous yeast *Phaffia rhodozyma* and its sexual form, *Xanthophyllomyces dendrorhous*. Appl. Environ. Microbiol..

[13] David-Palma M., Libkind D., Sampaio J.P. (2014). Global distribution, diversity hot spots and niche transitions of an astaxanthin-producing eukaryotic microbe. Mol. Ecol..

[14] Bolger A.M., Lohse M., Usadel B. (2014). Trimmomatic: A flexible trimmer for Illumina sequence data. Bioinformatics.

[15] Bushnell B., Rood J., Singer E. (2017). BBMerge—Accurate paired shotgun read merging via overlap. PLoS ONE.

[16] Bankevich A., Nurk S., Antipov D., Gurevich A.A., Dvorkin M., Kulikov A.S., Lesin V.M., Nikolenko S.I., Pham S., Prjibelski A.D. (2012). SPAdes: A new genome assembly algorithm and its applications to single-cell sequencing. J. Comput. Biol..

[17] Gurevich A., Saveliev V., Vyahhi N., Tesler G. QUAST: Quality Assessment Tool for Genome Assemblies. https://academic.oup.com/bioinformatics/article/29/8/1072/228832.

[18] Simpson J.T., Wong K., Jackman S.D., Schein J.E., Jones S.J.M., Birol I. (2009). ABySS: A parallel assembler for short read sequence data. Genome Res..

[19] Stanke M., Waack S. (2003). Gene prediction with a hidden Markov model and a new intron submodel—PubMed. Bioinformatics.

[20] Li L., Stoeckert C.J., Roos D.S. (2003). OrthoMCL: Identification of ortholog groups for eukaryotic genomes. Genome Res..

[21] Contreras-Moreira B., Vinuesa P. (2013). GET_HOMOLOGUES, a versatile software package for scalable and robust microbial pangenome analysis. Appl. Environ. Microbiol..

[22] Katoh K., Standley D.M. (2013). MAFFT multiple sequence alignment software version 7: Improvements in performance and usability. Mol. Biol. Evol..

[23] Criscuolo A., Gribaldo S. (2010). BMGE (Block Mapping and Gathering with Entropy): A new software for selection of phylogenetic informative regions from multiple sequence alignments. BMC Evol. Biol..

[24] Haubold B., Pfaffelhuber P., Domazet-Los˘o M., Wiehe T. (2009). Estimating mutation distances from unaligned genomes. J. Comput. Biol..

[25] Gremme G., Steinbiss S., Kurtz S. (2013). GenomeTools: A comprehensive software library for efficient processing of structured genome annotations. IEEE/ACM Trans. Comput. Biol. Bioinform..

[26] Bellora N., Moliné M., David-Palma M., Coelho M.A., Hittinger C.T., Sampaio J.P., Gonçalves P., Libkind D. (2016). Comparative genomics provides new insights into the diversity, physiology, and sexuality of the only industrially exploited tremellomycete: *Phaffia rhodozyma*. BMC Genom..

[27] Nguyen L.-T., Schmidt H.A., von Haeseler A., Minh B.Q. (2015). IQ-TREE: A fast and effective stochastic algorithm for estimating maximum-likelihood phylogenies. Mol. Biol. Evol..

[28] Kalyaanamoorthy S., Minh B.Q., Wong T.K.F., Von Haeseler A., Jermiin L.S. (2017). ModelFinder: Fast model selection for accurate phylogenetic estimates. Nat. Methods.

[29] Hoang D.T., Chernomor O., von Haeseler A., Minh B.Q., Vinh L.S. (2018). UFBoot2: Improving the ultrafast bootstrap approximation. Mol. Biol. Evol..

[30] Tamura K., Peterson D., Peterson N., Stecher G., Nei M., Kumar S. (2011). MEGA5: Molecular evolutionary genetics analysis using maximum likelihood, evolutionary distance, and maximum parsimony methods. Mol. Biol. Evol..

[31] Tusnady G.E., Simon I. (2001). The HMMTOP transmembrane topology prediction server. Bioinformatics.

[32] Mitchell A., Chang H.Y., Daugherty L., Fraser M., Hunter S., Lopez R., McAnulla C., McMenamin C., Nuka G., Pesseat S. (2015). The InterPro protein families database: The classification resource after 15 years. Nucleic Acids Res..

[33] Lin J., Hu J. (2013). SeqNLS: Nuclear localization signal prediction based on frequent pattern mining and linear motif scoring. PLoS ONE.

[34] Drozdetskiy A., Cole C., Procter J., Barton G.J. (2015). JPred4: A protein secondary structure prediction server. Nucleic Acids Res..

[35] Alva V., Nam S.Z., Soding J., Lupas A.N. (2016). The MPI bioinformatics Toolkit as an integrative platform for advanced protein sequence and structure analysis. Nucleic Acids Res..

[36] Lupas A., Van Dyke M., Stock J. (1991). Predicting coiled coils from protein sequences. Science.

[37] Hall T.A. (1999). BioEdit: A user-friendly biological sequence alignment editor and analysis program for Windows 95/98/NT. Nucleic Acids Symp. Ser..

[38] Kurtzman C.P., Fell J.W., Boekhout T., Robert V., Kurtzman C., Fell J., Boekhout T. (2011). Methods for isolation, phenotypic characterization and maintenance of yeasts. The Yeasts, a Taxonomic Study.

[39] Libkind D., Moliné M., García V., Fontenla S., Broock M. (2008). Characterization of a novel South American population of the astaxanthin producing yeast *Xanthophyllomyces dendrorhous* (*Phaffia rhodozyma*). Ind. Microbiol. Biotechnol..

[40] Libkind D., Moliné M., Tognetti C., Barredo J.-L. (2012). Isolation and selection of new astaxanthin producing strains of *Xanthophyllomyces dendrorhous*. Microbial Carotenoids from Fungi: Methods and Protocols.

[41] Liu X.Z., Wang Q.M., Göker M., Groenewald M., Kachalkin A.V., Lumbsch H.T., Millanes A.M., Wedin M., Yurkov A.M., Boekhout T. (2015). Towards an integrated phylogenetic classification of the *Tremellomycetes*. Stud. Mycol..

[42] Fell J.W., Boekhout T., Fonseca A., Scorzetti G., Statzell-Tallman A. (2000). Biodiversity and systematics of basidiomycetous yeasts as determined by large-subunit rDNA D1/D2 domain sequence analysis. Int. J. Syst. Evol. Microbiol..

[43] Hibbett D.S., Binder M., Bischoff J.F., Blackwell M., Cannon P.F., Eriksson O.E., Huhndorf S., James T., Kirk P.M., Lücking R. (2007). A higher-level phylogenetic classification of the *Fungi*. Mycol. Res..

[44] Libkind D., Čadež N., Opulente D.A., Langdon Q.K., Rosa C.A., Sampaio J.P., Gonçalves P., Hittinger C.T., Lachance M.A. (2020). Towards yeast taxogenomics: Lessons from novel species descriptions based on complete genome sequences. FEMS Yeast Res..

[45] Rokas A., Mead M.E., Steenwyk J.L., Raja H.A., Oberlies N.H. (2020). Biosynthetic gene clusters and the evolution of fungal chemodiversity. Nat. Prod. Rep..

[46] David-Palma M., Sampaio J.P., Gonçalves P. (2016). Genetic dissection of sexual reproduction in a primary homothallic basidiomycete. PLoS Genet..

[47] Coelho M.A., Bakkeren G., Sun S., Hood M.E., Giraud T., Heitman J., Howlett B.J., Crous P.W., Stukenbrock E.H., James T., Gow N.A.R. (2017). Fungal Sex: The Basidiomycota. The Fungal Kingdom.

[48] Devier B., Aguileta G., Hood M.E., Giraud T. (2009). Ancient trans-specific polymorphism at pheromone receptor genes in basidiomycetes. Genetics.

[49] McNeill J., Barrie F., Buck W., Demoulin V., Greuter W., Al E. (2012). International Code of Nomenclature for Algae, Fungi, and Plants (Melbourne Code). Regnum Vegetabile 154.

