# Supplementary material for: The Untapped Australasian Diversity of Astaxanthin-Producing Yeasts with Biotechnological Potential—Phaffia australis sp. nov. and Phaffia tasmanica sp. nov
Source: Microorganisms. 2020 Oct 24;8(11):1651. doi: 10.3390/microorganisms8111651 (PMC7692969; doi:10.3390/microorganisms8111651)
Supplement: Supplementary file 1 [file microorganisms-08-01651-s001.pdf]

Table S1: Localization of the genes encoding enzymes of the astaxanthin biosynthetic pathway in the draft genomes of *P. australis* and *P. tasmanica*.

**Gene**	**Contig (node) in *P. australis* PYCC 6859**	**Contig (node) in *P. tasmanica* PYCC 6858**
IDI - isopentenyl pyrophosphate isomerase	NODE_365_length_5288_cov_17.8917_ID_729	NODE_103_length_61302_cov_20.3239_ID_205
crtE - geranylgeranyl pyrophosphate synthase	NODE_17_length_156497_cov_17.9011_ID_33	NODE_17_length_123067_cov_19.576_ID_33
crtYB - phytoene synthase and lycopene cyclase	NODE_304_length_11423_cov_19.2003_ID_607	NODE_183_length_38676_cov_19.0123_ID_365
crtI - phytoene desaturase	NODE_59_length_89386_cov_21.141_ID_117	NODE_27_length_106799_cov_20.9276_ID_53
crtS - astaxanthin synthetase	NODE_63_length_84228_cov_17.8916_ID_125	NODE_273_length_20701_cov_20.2642_ID_545
crtR - cytochrome P450 reductase	NODE_182_length_35959_cov_18.3017_ID_363	NODE_137_length_52196_cov_20.5677_ID_273

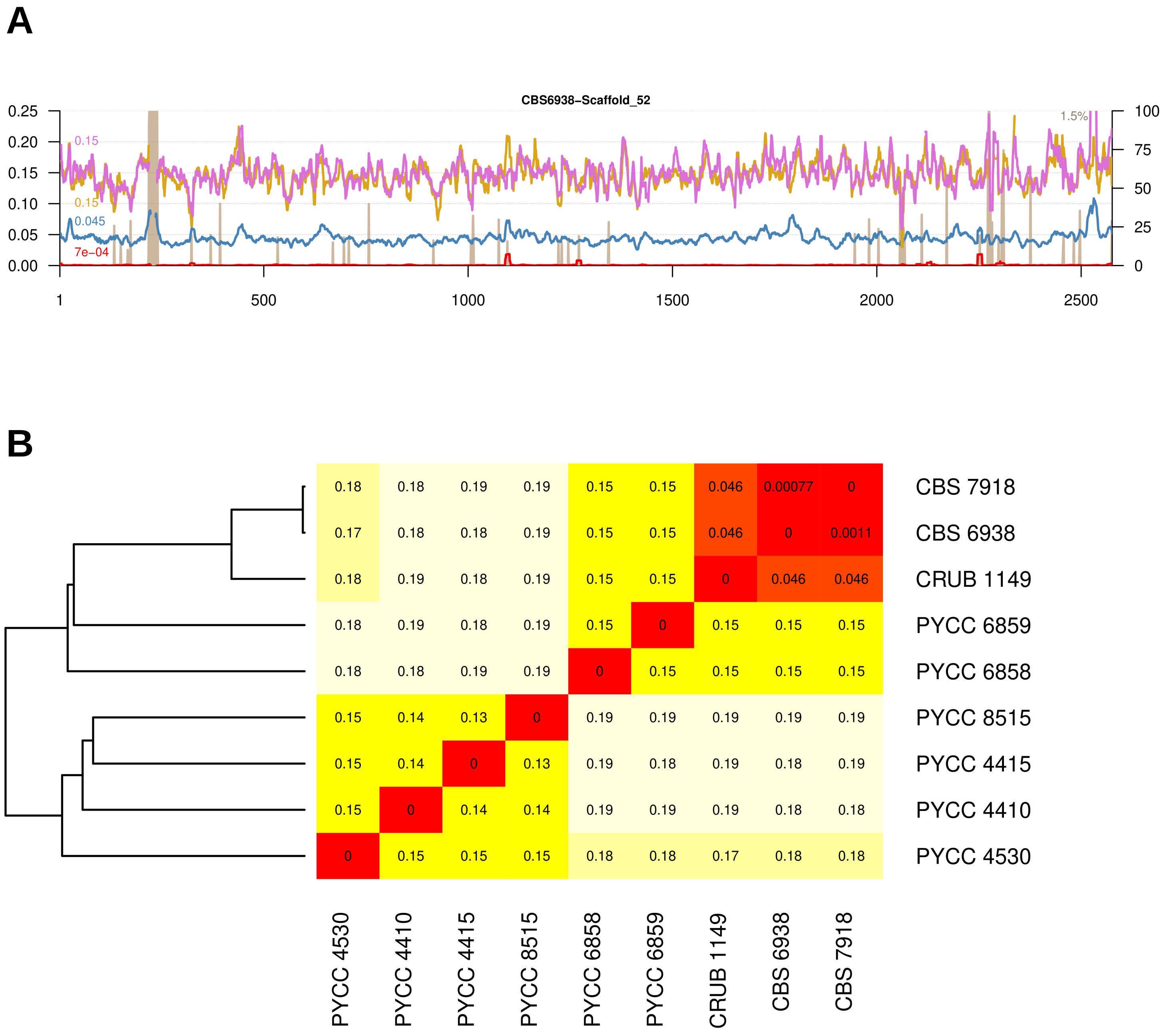

Figure S1. (A) Sequence similarity between the new species *Phaffia australis* and *P. tasmanica* and *P. rhodozyma* along the longest scaffold (scaffold 52) of *P. rhodozyma* CBS 6938. The left scale represents the divergence measured by a sliding window approach to *P. rhodozyma* CBS 7918 (red), *P. rhodozyma* CRUB 1149 (blue), *P. australis* PYCC 6859 (yellow) and *P. tasmanica* PYCC 6858 (magenta). The mean divergence value for each strain is shown using the color-codes indicated above. The right Y scale represents the percentage of Ns in the reference sequence, which are indicated as grey bars (1.5% of the scaffold sequence corresponded to Ns). (B) Genomic sequence divergence using an alignment-free method. Heat map and clustering with pairwise distances (Kr values) between the draft genomes of *P. rhodozyma* CBS 7918, CRUB 1149 and CBS 6938, *P. tasmanica* PYCC 6858, *P. australis* PYCC 6859, *C. bisporidii* PYCC 4415, *C. macerans* PYCC 8515, *C. capitatum* PYCC 4530 and *C. ferigula* PYCC 4410

A)

```
                            10        20        30        40        50        60        70
                    ....|....|....|....|....|....|....|....|....|....|....|....|....|....|
crtE_CBS 7918       MDYANILTAIPLEFTPQDDIVLLEPYHYLGKNPGKEIRSQLIEAFNYWLDVKKEDLEVIQNVVGMLHTAS
crtE_PYCC 6859      MDYANILTAVPLEFTPQDDIVLLEPYHYLGKNPGKEIRSQLIEAFNFWLDVKKEDLEVIQNVVGMLHTAS
crtE_PYCC 6858      MDYANILTAIPLEFTPQDDIVLLEPYHYLGKNPGKEIRSQLIEAFNFWLDVKKEDLEVIQNVVGMLHTAS
Clustal Consensus   *********:************************************:***********************

                            80        90       100       110       120       130       140
                    ....|....|....|....|....|....|....|....|....|....|....|....|....|....|
crtE_CBS 7918       LLMDDVEDSSVLRRGSPVAHLIYGIPQTINTANYVYFLAYQEIFKLRPTPIPMPVIPPSSASLQSSVSSA
crtE_PYCC 6859      LLMDDVEDSSVLRRGSPVAHLIYGIPQTINTANYVYFLAYQEIFKLRPTPTPTPVIPPSSASLQSSVSSA
crtE_PYCC 6858      LLMDDVEDSSVLRRGSPVAHLIYGIPQTINTANYVYFLAYQEIFKLRPTPIPVMPPSSASLQSSVSSA
Clustal Consensus   ************************************************** * **:**************

                           150       160       170       180       190       200       210
                    ....|....|....|....|....|....|....|....|....|....|....|....|....|....|
crtE_CBS 7918       SSSSSASSENGGTSTPNSQIPFSKDTYLDKVITDEMLSLHRGQGLELFWRDSLTCPSEEEYVKMVLGKTG
crtE_PYCC 6859      SSSSSTSSDHGGTSTPNSQIPFSKDTYLDKVITDEMLSLHRGQGLELFWRDSLTCPSEEEYVKMVLGKTG
crtE_PYCC 6858      SSFSSISSENGGTSTPNSQIPFSKDTYLDKVITDEMLSLHRGQGLELFWRDSLTCPSEEEYVKMVLGKTG
Clustal Consensus   ** ** **::************************************************************

                           220       230       240       250       260       270       280
                    ....|....|....|....|....|....|....|....|....|....|....|....|....|....|
crtE_CBS 7918       GLFRIAVRLMMAKSECDIDFVQLVNLISIYFQIRDDYMNLQSSEYAHNKNFAEDLTEGKFDTLQKKSTSP
crtE_PYCC 6859      GLFRIAVRLMMAKSECDIDFVQLVNLISIYFQIRDDYMNLQSSEYAHNKNFAEDLTEGKFNTLQKKSTSP
crtE_PYCC 6858      GLFRIAVRLMMAKSECDIDFIQLVNLISIYFQIRDDYMNLQSSEYAHNKNFAEDLTEGKFNTLQKKSTSP
Clustal Consensus   ********************:***************************************:*********

                           290       300       310       320       330       340       350
                    ....|....|....|....|....|....|....|....|....|....|....|....|....|....|
crtE_CBS 7918       EILHHCVNYMRTETHSFEYTQEVLNTLSGALERELGRLQGEFAEANSRMDLGDVDSEGRTGKNVKLEAIL
crtE_PYCC 6859      EILHHCVNYMRTETHSFEYTRDVLNTLSGALERELDRLQGEFAEFGSRMDIGDLDSEGKAGKNVKLETIL
crtE_PYCC 6858      EILHHCVNYMRTETNSFEYTREVLNTLSGALERELNRLQKEFAEAGSRMDIGDEEAEEKAGKNVKLEAIL
Clustal Consensus   **************:*****::*************.*** **** .****:** ::* ::*******:**


                    ....|...
crtE_CBS 7918       KKLADIPL
crtE_PYCC 6859      KKLADIPL
crtE_PYCC 6858      KRLADIPL
Clustal Consensus   *:******
```

B)

```
                            10        20        30        40        50        60        70
                    ....|....|....|....|....|....|....|....|....|....|....|....|....|....|
Idi_CBS 7918        MSMPNIVPPAEVRTEGLSLEEYDEEQVRLMEERCILVNPDDVAYGEASKKTCHLMSNINAPKDLLHRAFS
Idi_PYCC 6859       MSMPNIVPPAEIRTDGLSLEEYDEEQVRLMEERCILVNPDDVAYGEASKKTCHLMSNINAPKDLLHRAFS
Idi_PYCC 6858       MSKPSVATPAEIRTDGLSLEEYDEEQVRLMEERCILVNPDDVAYGEASKKICHLMSNINAPKDLLHRAFS
Clustal Consensus   ** *.:..***:**:*********************************** *******************

                            80        90       100       110       120       130       140
                    ....|....|....|....|....|....|....|....|....|....|....|....|....|....|
Idi_CBS 7918        VFLFRPSDGALLLQRRADEKITFPGMWTNTCCSHPLSIKGEVEEENQIGVRRAASRKLEHELGVPTSSTP
Idi_PYCC 6859       VFLFRPSDGALLLQRRADEKITFPGMWTNTCCSHPLSIKGEVEEENQIGVRRAASRKLEHELGVPTSATP
Idi_PYCC 6858       VFLFRPSDGALLLQRRADEKITFPGMWTNTCCSHPLSIKGEIEEENQMGVRRAASRKLEHELGVPTSSTP
Clustal Consensus   *****************************************:*****:*******************:**

                           150       160       170       180       190       200       210
                    ....|....|....|....|....|....|....|....|....|....|....|....|....|....|
Idi_CBS 7918        PDSFTYLTRIHYLAPSDGLWGEHEIDYILFSTTPTEHTGNPNEVSDTRYVTKPELQAMFEDESNSFTPWF
Idi_PYCC 6859       PDSFTYLTKIHYLAPSDGLWGEHEIDYILFSTTPTEHTGNPNEVSDTRYVTKPELQAMFEDESNSFTPWF
Idi_PYCC 6858       PDSFTYLTRIHYLAPSDGLWGEHEIDYILFSTTPTEHTGNPNEVSDTRYVTKSELQTMFEDESNSFTPWF
Clustal Consensus   ********:*******************************************.***:*************

                           220       230       240       250
                    ....|....|....|....|....|....|....|....|.
Idi_CBS 7918        KLIARDFLFGWWDQLLARRNEKGEVDAKSLEDLSDNKVWKM
Idi_PYCC 6859       KLIARDFLFGWWDQLLARRNEKGEVDAKSLEDLSDDKVWKM
Idi_PYCC 6858       KLIARDFLFGWWDELLARRNEKGEVDAKSLEYLSDDKVWKM
Clustal Consensus   *************:***************** ***:*****
```

C)

```
                       10        20        30        40        50        60        70
                   ....|....|....|....|....|....|....|....|....|....|....|....|....|....|
CrtYB_CBS 7918     MTALAYYQIHLIYTLPILGLLGLLTSPILTKFDIYKISILVFIAFSATTPWDSWIIRNGAWTYPSAESGQ
CrtYB_PYCC 6859    MTALAYYQIHLIYTLPILGLLGLLTSPILTKFDIYKISILVFIAFTATTPWDSWIIRNGAWTYPSAETGQ
CrtYB_PYCC 6858    MAALAYYQIHLIYTLPILGLLLLLTSPILTRFDIYKVSILVFIAFSATTPWDSWIIRNGAWTYPSAESGQ
Clustal Consensus  *:******************* ********:*****:********:*********************:**

                       80        90       100       110       120       130       140
                   ....|....|....|....|....|....|....|....|....|....|....|....|....|....|
CrtYB_CBS 7918     GVFGTFLDVPYEEYAFFVIQTVITGLVYVLATRHLLPSLALPKTRSSALSLALKALIPLPIIYLFTAHPS
CrtYB_PYCC 6859    GVLGTFLDVPYEEYAFFIIQTVITGLVYVLATRHLLPSLALPKTRSSARSLALKALIPLPIIYLFTAHPS
CrtYB_PYCC 6858    GVLGTFLDVPYEEYAFFIIQTVITGLVYVLATRHLLPSLALPKTRSSARSLALKALIPLPIIYLFAVHPS
Clustal Consensus  **:**************:****************************** ****************:.***

                      150       160       170       180       190       200       210
                   ....|....|....|....|....|....|....|....|....|....|....|....|....|....|
crtYB_CBS 7918     PSPDPLVTDHYFYMRALSLLITPPTMLLAALSGEYAFDWKSGRAKSTIAAIMIPTVYLIWVDYVAVGQDS
CrtYB_PYCC 6859    PSPDPLVTDHYFYMRALSLLITAPTMLLATLSGEYAFDWRSGRAKSTLAAITIPTVYLIWVDYVAVGQES
CrtYB_PYCC 6858    PSSDPLVTDHYFYMRALSLLTTPPTMLLATLSGEYAFSWRSGRAKSTIAAIMVPTVYLIWVDYIAVGQES
Clustal Consensus  **.***************** *.******:*******.*:*******:*** :**********:****:*

                      220       230       240       250       260       270       280
                   ....|....|....|....|....|....|....|....|....|....|....|....|....|....|
crtYB_CBS 7918     WSINDEKIVGWRLGGVLPIEEAMFFLLTNLMIVLGLSACDHTQALYLLHGRTIYGNKKMPSSFPLITPPV
CrtYB_PYCC 6859    WSINDEKIVGWRLGGVLPIEEALFFLLTNLMIVLGLSACDHTQALYLLHGRTIYGKKQMPLSFPLVMPPV
CrtYB_PYCC 6858    WSINDEKIVGWRFGGVLPIEEALFFLLTNLMIVLGLSACDHTQALYLLHGRTIYGKKKMPSSFPLITPPV
Clustal Consensus  ************:*********:********************************:*:** ****: ***

                      290       300       310       320       330       340       350
                   ....|....|....|....|....|....|....|....|....|....|....|....|....|....|
crtYB_CBS 7918     LSLFFSSRPYSSQPKRDLELAVKLLEKKSRSFFVASAGFPSEVRERLVGLYAFCRVTDDLIDSPEVSSNP
CrtYB_PYCC 6859    LSLFFSSRPYSSQPKRDLELAVKILEKKSRSFFVASAGFPCEVRERLVGLYAFCRVTDDLIDSPDMSSNP
CrtYB_PYCC 6858    LSLFFSSRPYSPQPKRDLELAVKLLEKKSRSFFVASAGFPSEVRERLVGLYAFCRVTDDLIDSPEVSSNP
Clustal Consensus  ***********.***********:****************.***********************::****

                      360       370       380       390       400       410       420
                   ....|....|....|....|....|....|....|....|....|....|....|....|....|....|
crtYB_CBS 7918     HATIDMVSDFLTLLFGPPLHPSQPDKILSSPLLPPSHPSRPTGMYPLPPPPSLSPAELVQFLTERVPVQY
CrtYB_PYCC 6859    NDTIDMISDFLTLIFGPSLHPSHRDETLSSTLLPPTHPSRPTGLYPLSPPPSFTSVELDQFLTKRVPVQY
CrtYB_PYCC 6858    HDTIRMISDFLTLLFGPPLHHSQSDKSISSDLLPPPHPSRPTGMYPLSPPPSISTVDLNQFLTERVPVDS
Clustal Consensus  : ** *:******:***.** *: *: :** ****.*******:***.****::..:* ****:****:

                      430       440       450       460       470       480       490
                   ....|....|....|....|....|....|....|....|....|....|....|....|....|....|
crtYB_CBS 7918     HFAFRLLAKLQGLIPRYPLDELLRGYTTDLIFPLSTEAVQARKTPIETTADLLDYGLCVAGSVAELLVYV
CrtYB_PYCC 6859    HFAFRLLAKLQGLIPRYPLDELLRGYTADLAFPLSTESVEARKTPIKTTADLLDYGLCVAGSVAELLVYV
CrtYB_PYCC 6858    HFAFRLLAKLQGLIPRYPLDELLRGYTTDLAFPLSTKAIEARKAPIETTADLLDYSLCVAGSVAELLVYV
Clustal Consensus  ***************************:** *****::::***:*:********.***************

                      500       510       520       530       540       550       560
                   ....|....|....|....|....|....|....|....|....|....|....|....|....|....|
crtYB_CBS 7918     SWASAPSQVPATIEEREAVLVASREMGTALQLVNIARDIKGDATEGRFYLPLSFFGLRDESKLAIPTDWT
CrtYB_PYCC 6859    SWASAQSQVPATVEEREAVLMASREMGTALQLVNIARDIKGDATEGRCYLPLSFFGLRDESKLAIPIDWE
CrtYB_PYCC 6858    SWASAPDQVPATIEGRETVLVASREMGSALQLVNIARDIKGDAEEGRLYLPLSFFGLREESKIAIPIDWT
Clustal Consensus  ***** .*****:* **:**:******:*************** *** **********:***:*** **

                      570       580       590       600       610       620       630
                   ....|....|....|....|....|....|....|....|....|....|....|....|....|....|
crtYB_CBS 7918     EPRPQDFDKLLSLSPSSTLPSSNASESFRFEWKTYSLPLVAYAEDLAKHSYKGIDRLPTEVQAGMRAACA
CrtYB_PYCC 6859    KSRPQDFDELLSLSPASTSSSPNTSGNLRFEWKTYSLPLIAYAEDLAKHSYKGIDLLPTEVRAGMRAACA
CrtYB_PYCC 6858    KPRPQDFDELLSLSPSSTSSSPKILESFQFGWKMYSLPLIAYAEDLAKHSYKGIDRLPTEVRAGMRAACA
Clustal Consensus  :.******:******:** .*.:   .::* ** *****:*************** *****:********

                      640       650       660       670
                   ....|....|....|....|....|....|....|....|...
crtYB_CBS 7918     SYLLIGREIKVVWKGDVGERRTVAGWRRVRKVLSVVMSGWEGQ
CrtYB_PYCC 6859    SYLLIGREIKVVWKGDVGERRTVAGWRRVQKVLSVVMNGWEGQ
CrtYB_PYCC 6858    SYLLIGREIKVVWKGDVGERRTVAGWRRVRRVLSVVVNGWEGQ
Clustal Consensus  *****************************::*****:.*****
```

D)

```
                           10        20        30        40        50        60        70
                   ....|....|....|....|....|....|....|....|....|....|....|....|....|....|
CrtI_CBS 7918      MGKEQDQDKPTAIIVGCGIGGIATAARLAKEGFQVTVFEKNDYSGGRCSLIERDGYRFDQGPSLLLLPDL
CrtI_PYCC 6859     MGKEQDQDKPTAIIVGCGIGGIATAARLAKEGFQVTVFEKNDYSGGRCSLIERDGYRFDQGPSLLLLPDL
CrtI_PYCC 6858     MGKEQEQNKPTAIIVGCGIGGIFTAARLAKEGFQVTVFEKNDYSGGRCSLIERDGYRFDQGPSLLLLPDL
Clustal Consensus  *****:*:************** ***********************************************

                           80        90       100       110       120       130       140
                   ....|....|....|....|....|....|....|....|....|....|....|....|....|....|
CrtI_CBS 7918      FKQTFEDLGEKMEDWVDLIKCEPNYVCHFHDEETFTLSTDMALLKREVERFEGKDGFDRFLSFIQEAHRH
CrtI_PYCC 6859     FKQTFEDLGEKMEDWVDLIKCEPNYVCHFHDDETFTLSTDMALLKREVERFEGKDGFDRFLSFIQEAHRH
CrtI_PYCC 6858     FKQTYEDLGEKMEDWVDLIKCEPNYVCHFHDEETFTLSTDLALLKREVERFEGKDGFDRFLSFIQEAHRH
Clustal Consensus  ****:**************************:********:*****************************

                          150       160       170       180       190       200       210
                   ....|....|....|....|....|....|....|....|....|....|....|....|....|....|
CrtI_CBS 7918      YELAVVHVLQKNFPGFAAFLRLQFIGQILALHPFESIWTRVCRYFKTDRLRRVFSFAVMYMGQSPYSAPG
CrtI_PYCC 6859     YELAVVHVLQKNFPGFAAFLRLQFIGQILALHPFESIWTRVCRYFKTDRLRRVFSFAVMYMGQSPYSAPG
CrtI_PYCC 6858     YELAVVHVLQKNFPGFAAFLRLQFIGQILALHPFESIWTRVCRYFKTDRLRRVFSFAVMYMGQSPYSAPG
Clustal Consensus  **********************************************************************

                          220       230       240       250       260       270       280
                   ....|....|....|....|....|....|....|....|....|....|....|....|....|....|
CrtI_CBS 7918      TYSLLQYTELTEGIWYPRGGFWQVPNTLLQIVKRNNPSAKFNFNAPVSQVLLSPAKDRATGVRLESGEEH
CrtI_PYCC 6859     TYSLLQYTELTEGIWYPRGGFWQVPNTLLQIVKRNNPSAKFNFNAPVSKVLLSPSKDRATGVRLESGEEH
CrtI_PYCC 6858     TYSLLQYTELTEGIWYPRGGFWQVPNTLLQIVKRNNPSAKFNFNTPVSRVLLSPAKDRAIGVRLESGEEH
Clustal Consensus  ********************************************:***:*****:**** **********

                          290       300       310       320       330       340       350
                   ....|....|....|....|....|....|....|....|....|....|....|....|....|....|
CrtI_CBS 7918      HADVVIVNADLVYASEHLIPDDARNKIGQLGEVKRSWWADLVGGKKLKGSCSSLSFYWSMDRIVDGLGGH
CrtI_PYCC 6859     HADVVIVNADLVYASEHLIPDDARNKSGQLGEVKRSWWADLVGGKKLKGSCSSLSFYWSMDRIVDGLGGH
CrtI_PYCC 6858     YADVVIVNADLVYASEHLIPDDARNKTGQLGQVKRSWWADLVGGKKLKGSCSSLSFYWSMDRIVDGLGGH
Clustal Consensus  :************************* ****:**************************************

                          360       370       380       390       400       410       420
                   ....|....|....|....|....|....|....|....|....|....|....|....|....|....|
CrtI_CBS 7918      NIFLAEDFKGSFDTIFEELGLPADPSFYVNVPSRIDPSAAPEGKDAIVILVPCGHIDASNPQDYNKLVAR
CrtI_PYCC 6859     NIFLAEDFKGSFDTIFEELGLPADPSFYVNVPSRIDPSAAPEGKDAIVILVPCGHIDTSNPQDYKKLVAR
CrtI_PYCC 6858     NIFLAEDFKGSFDTIFEELGLPADPSFYVNVPSRIDPSAAPEGKDAIVILVPCGHINTSNPQDYNKLVAR
Clustal Consensus  ********************************************************::******:*****

                          430       440       450       460       470       480       490
                   ....|....|....|....|....|....|....|....|....|....|....|....|....|....|
CrtI_CBS 7918      ARKFVIQTLSAKLGLPDFEKMIVAEKVHDAPSWEKEFNLKDGSILGLAHNFMQVLGFRPSTRHPKYDKLF
CrtI_PYCC 6859     ARKFVIETLSAKLGIPDFEKMIVAEKVHDAPSWEKEFNLKDGSILGLAHNFMQVLGFRPSTRHPKYDKLF
CrtI_PYCC 6858     AREFVIQTLSAKLGIPDFEKMIVAEKVHDAHSWEKEFNLKDGSILGLAHNFMQVLGFRPSTRHPKYDKLF
Clustal Consensus  **:***:*******:*************** ***************************************

                          500       510       520       530       540       550       560
                   ....|....|....|....|....|....|....|....|....|....|....|....|....|....|
CrtI_CBS 7918      FVGASTHPGTGVPIVLAGAKLTANQVLESFDRSPAPDPNMSLSVPYGKPLKSNGTGIDSQVQLKFMDLER
CrtI_PYCC 6859     FVGASTHPGTGVPIVLAGAKLTANQVLESFNRPPAPDPDMSLSVPYGKPLKSKGTGIDAQVDLKPMDLEK
CrtI_PYCC 6858     FVGASTHPGTGVPIVLAGAKLTANQVLESFDRPPAPDPNMSLSVPYGKPLKSNGTGIDTQIQLKSMDLER
Clustal Consensus  ******************************:*.*****:*************:*****:*::** ****:

                          570       580
                   ....|....|....|....|..
CrtI_CBS 7918      WVYLLVLLIGAVIARSVGVLAF
CrtI_PYCC 6859     WVYFFMLLIGAAIARFYGVLNF
CrtI_PYCC 6858     WVYLLLVLVAVFVVRFIGALP-
Clustal Consensus  ***::::*:.. :.*  *.*
```

E)

```
                           10        20        30        40        50        60        70
                   ....|....|....|....|....|....|....|....|....|....|....|....|....|....|
CrtR_CBS 7918      MATLSDLVILLLGALLALGFYNKDRLLGSSSSSASTTSGSSAATANGSKPTDSSGNGNAFKGDPRDFVAR
CrtR_PYCC 6859     MATLSDLVILLLGALLALGFFYKDRLLGSSTSSTATASTSSAATTNGTKLAESNGNGHAFKGDPRDFVER
CrtR_PYCC 6858     MASLSDLVIILLGALLALGLYNKDRLLGS-KSSSATTTASTATTIDGTKSAESNGNGHIFKGDPRDFVAR
Clustal Consensus  **:******:*********:: ******* .**::*:: *:*:* :*:* ::*.***: ********* *

                           80        90       100       110       120       130       140
                   ....|....|....|....|....|....|....|....|....|....|....|....|....|....|
CrtR_CBS 7918      MKDQKKRLAVFYGSQTGTAEEYATRIAKEAKSRFGVSSLVCDIEEYDFEKLDQVPEDCAIVFCMATYGEG
CrtR_PYCC 6859     MKDQKKRLAVFYGSQTGTAEEYATRIAKEAKSRFGVSSLVCDIEEYDFEKLDQVPEDCAIVFCMATYGEG
CrtR_PYCC 6858     MIDQKKRLAVFYGSQTGTAEEYATRIAKEAKSRFGVSSLVCDIEEYDFEKIDQVPEDCAIVFCMATYGEG
Clustal Consensus  * ************************************************:*******************

                          150       160       170       180       190       200       210
                   ....|....|....|....|....|....|....|....|....|....|....|....|....|....|
CrtR_CBS 7918      EPTDNAVQFIEMISQDDPEFSEGSTLDGLKYVVFGLGNKTYEQYNVVGRQLDARLTALGATRVGERGEGD
CrtR_PYCC 6859     EPTDNAIQFTEMINQDDPEFSNGSTLDGLKYVVFGLGNKTYEQYNVVGRQLDARLTALGATRIGERGEGD
CrtR_PYCC 6858     EPTDNAVQFIEMINQDDPEFSNGSTLDGLKYVVFGLGNKTYEQYNVIGRQLDSRLTSLGATRIGERGEGD
Clustal Consensus  ******:** ***.*******:************************:*****:***:*****:*******

                          220       230       240       250       260       270       280
                   ....|....|....|....|....|....|....|....|....|....|....|....|....|....|
CrtR_CBS 7918      DDKSMEEDYLAWKDDMFAALATTLSFEEGASGETPDFVVTEVPNHPIEKVFQGELSSRALLGSKGVHDAK
CrtR_PYCC 6859     DDKSMEEDYLAWKDDMFAALAAKLSFEEGASGETPDFVVTEVPNHPIEKVFQGELSSRALFGSKGVHDAK
CrtR_PYCC 6858     DDKSMEEDYLAWKDDMFAALATTLSFEEGASGETPDFVVTEVPSHPIEKVFQGELSSRALLGSKGVHDAK
Clustal Consensus  *********************:.********************.****************:*********

                          290       300       310       320       330       340       350
                   ....|....|....|....|....|....|....|....|....|....|....|....|....|....|
CrtR_CBS 7918      NPYASPVLACRELFTGGDRNCIHLEFDITGSGITYQTGDHVAVWPSNPDVEVERLLAVLGLTSPEKRRMI
CrtR_PYCC 6859     NPYASPVLTCRELFTGGDRNCIHLEFDITGSGITYQTGDHVAVWPSNPDVEVERLLAVLGLTLPEKRHMI
CrtR_PYCC 6858     NPYASPVLACRELFTGGDRNCIHLEFDITGSGITYQTGDHVAVWPSNPDVEVERLLAVLGLTSPEKRHMI
Clustal Consensus  ********:***************************************************** ****:**

                          360       370       380       390       400       410       420
                   ....|....|....|....|....|....|....|....|....|....|....|....|....|....|
CrtR_CBS 7918      IQVVSLDPTLAKVPFPTPTTYDAVFRHYLDISAVASRQTLAVLAKYAPSEQAAEFLTRLGTDKQAYHTEV
CrtR_PYCC 6859     IQVVSLDPTLAKVPFPTPTTYDAVFRHYLDVSAVASRQTLAVLAKYAPSEEAAQFLTRLGTDKQAYHTEV
CrtR_PYCC 6858     IQVLSLDPTLAKVPFPTPTTYDAVFRHYLDVSAVASRQTLAILAKYAPSEQAAEFLTRLGTDKQAYHTEV
Clustal Consensus  ***:**************************:**********:********:**:****************

                          430       440       450       460       470       480       490
                   ....|....|....|....|....|....|....|....|....|....|....|....|....|....|
CrtR_CBS 7918      VGGHLRLAEVLQLAAGNDITVMPTAENTTVWNIPFDHVVSDVSRLQPRFYSISSSPKLHPNSIHVTAVIL
CrtR_PYCC 6859     VGGHLRLAEVLQLAAGNDIAVMPTAENTTVWNIPFDHVVSDISRLQPRFYSISSSPKLHPNSIHVTAVIL
CrtR_PYCC 6858     IGGHLRLAEVLQLAAGNDITVMPTAENTTMWNIPFDHIVSDVSRLQPRFYSISSSPKLYPNSIHVTAVIL
Clustal Consensus  :******************:*********:*******:***:****************:***********

                          500       510       520       530       540       550       560
                   ....|....|....|....|....|....|....|....|....|....|....|....|....|....|
CrtR_CBS 7918      KYESQATDRHPARWVFGLGTNYLLNVKQAANNETTPMISDGQDDVPEHVSAPKYTLEGPRGSYKHDDQLF
CrtR_PYCC 6859     KYSSQATDGHPARWVFGLGTNYLLNVKQAANNETTPMISNGQDDVPEHVSSPKYTLEGPRGSYKHNDQLF
CrtR_PYCC 6858     KYSSQASDRHPARWVFGLGTNYLLNVKQAANEETTPMISDGQDDIPEHVSAPKYTLEGPRGSYKHDDKLF
Clustal Consensus  **.***:* **********************:*******:****:*****:**************:*:**

                          570       580       590       600       610       620       630
                   ....|....|....|....|....|....|....|....|....|....|....|....|....|....|
CrtR_CBS 7918      KVPIHVRRSTFRLPTSPKIPVIMIGPGTGVAPFRGFIQERIALARRSIAKNGPDALADWAPIYLFYGSRD
CrtR_PYCC 6859     KVPIHVRRSTFRLPTSPKIPVIMIGPGTGVAPFRGFIQERIALARRSIAKNGPDALADWAPIYLFYGSRD
CrtR_PYCC 6858     KVPIHVRRSTFRLPTSPKIPVIMIGPGTGVAPFRGFIQERIALARRSIAKNGPDALADWAPIYLFYGSRD
Clustal Consensus  **********************************************************************

                          640       650       660       670       680       690       700
                   ....|....|....|....|....|....|....|....|....|....|....|....|....|....|
CrtR_CBS 7918      EQDFLYAEEWPAYEAELQGKFKIHVAFSRSGPRKPDGSKIYVQDLLWDQKEVIKSAIVEKRASVYICGDG
CrtR_PYCC 6859     EQDFLYADEWPAYEAELQGKFKIHVAFSRSGPRKPDGSKIYVQDLLWDQKEAIKSAIVEKRASVYICGDG
CrtR_PYCC 6858     EQDFLYADEWPAYEAELQGKFKIHVAFSRSGPRKPDGSKIYVQDLLWEQKEAIKSAIVEKRASVYICGDG
Clustal Consensus  *******:***************************************:***.******************

                          710       720       730       740
                   ....|....|....|....|....|....|....|....|....|.
CrtR_CBS 7918      RNMSKDVEQKLAAMLAESKNGSAAVEGAAEVKSLKERSRLLMDVWS
CrtR_PYCC 6859     RNMSKDVEQKLAAMLAESKNGSAAVEGAAELKSLKERSRLLMDVWS
CrtR_PYCC 6858     RNMSKDVEQKLAAMLAESKNGSAAVEGAAEVKSLKERSRLLMDVWS
Clustal Consensus  ******************************:***************
```

F)

```
                           10        20        30        40        50        60        70
                   ....|....|....|....|....|....|....|....|....|....|....|....|....|....|
CrtS_CBS 7918      MFILVLLTGALGLAAFSWASIAFFSLYLAPRRSSLYNLQGPNHTNYFTGNFLDILSARTGEEHAKYREKY
CrtS_PYCC 6859     MSILVLLTGVLGLAAFSWAFIAFFSLYLAPRQSSLYNLQGPDHTNYFTGNFLDILSARTGEEHAKYREKY
CrtS_PYCC 6858     MSILILFTGALGLTAFFWASIAFFSLYLAPRRSSLYNLQGPDHTNFLTGNFLEILSARTGEEHAKYREKY
Clustal Consensus  * **:*:**.***:** ** ***********:*********:***::*****:*****************

                           80        90       100       110       120       130       140
                   ....|....|....|....|....|....|....|....|....|....|....|....|....|....|
CrtS_CBS 7918      GSTLRFAGIAGAPVLNSTDPKVFNHVMKEAYDYPKPGMAARVLRIATGDGVVTAEGEAHKRHRRIMIPSL
CrtS_PYCC 6859     GSTLRFAGIAGTPVLNSTDPKVFNHVMKEAYDYPKPGMAARVLRIATGDGVVTAEGEAHKRHRRIMIPSL
CrtS_PYCC 6858     GSTLRFAGIAGMPVLNSTDPKVFNHVMKEAYDYPKPGMAARVLRIATGDGVVTAEGEAHKRHRRIMIPSL
Clustal Consensus  *********** **********************************************************

                          150       160       170       180       190       200       210
                   ....|....|....|....|....|....|....|....|....|....|....|....|....|....|
CrtS_CBS 7918      SAQAVKSMVPIFLEKGMELVDKMMEDAAEKDMAVGESAGEKKATRLETEGVDVKDWVGRATLDVMALAGF
CrtS_PYCC 6859     SAQAVKSMVPIFLEKGMELVDKMMEDAAEKDMAVGDSAGEKKATRLETEGVDVKDWVGRATLDVMALAGF
CrtS_PYCC 6858     SAQAVKSMVPIFLEKGMELVDKMMEDAAEKDMTVGESIGEKKVTRLDVEGVDVKDWVGRATLDVMALAGF
Clustal Consensus  ********************************:**:* ****.***:.**********************

                          220       230       240       250       260       270       280
                   ....|....|....|....|....|....|....|....|....|....|....|....|....|....|
CrtS_CBS 7918      DYKSDSLQNKTNELYVAFVGLTDGFAPTLDSFKAIMWDFVPYFRTMKRRHEIPLTQGLAVSRRVGIELME
CrtS_PYCC 6859     DYKSDSLQNKTNELYVAFVGLTDGFAPTLDSFMAIMWDFIPYFRTMKRKHEIPLTQGLAVSRRVGIELME
CrtS_PYCC 6858     DYKSDSLQNKFNELYVAFVGLTDGFAPTFDSAMAIMWDFIPFFRTMKRRREIPLTQGLAVSRRVGIELME
Clustal Consensus  ********** *****************:**  ******:*:******::********************

                          290       300       310       320       330       340       350
                   ....|....|....|....|....|....|....|....|....|....|....|....|....|....|
CrtS_CBS 7918      QKKQAVLGSASDQAVDKKDVQGRDILSLLVRANIAANLPESQKLSDEEVLAQISNLLFAGYETSSTVLTW
CrtS_PYCC 6859     QKKQAVLDSSSDQAVDKKDVQGRDILSLLVRANIAANLPESQKLSDEEVLAQISNLLFAGYETSSTVLTW
CrtS_PYCC 6858     QKKQSVLGSSSDQAVDKKDVEGRDILSLLVRANIAANLPESQKLSDEEVLAQISNLLFAGYETSSTVLTW
Clustal Consensus  ****:**.*:**********:*************************************************

                          360       370       380       390       400       410       420
                   ....|....|....|....|....|....|....|....|....|....|....|....|....|....|
CrtS_CBS 7918      MFHRLSEDKAVQDKLREEICQIDTDMPTLDELNALPYLEAFVKESLRLDPPSPYANRECLKDEDFIPLAE
CrtS_PYCC 6859     MFHRLSEDKAVQDRLREEICQINTDTPTLDELNALPYLEAFVKESLRLDPPSPYANRECLKDEDFIPLAE
CrtS_PYCC 6858     MFHRLSEDKTVQDKLREEICQIDTDMPTLDELNALPYLEAFVKESLRLDPPSPYANRECLKDEDFIPLAE
Clustal Consensus  *********:***:********:** ********************************************

                          430       440       450       460       470       480       490
                   ....|....|....|....|....|....|....|....|....|....|....|....|....|....|
CrtS_CBS 7918      PVIGRDGSVINEVRITKGTMVMLPLFNINRSKFIYGEDAEEFRPERWLEDVTDSLNSIEAPYGHQASFIS
CrtS_PYCC 6859     PVIGRDGSVINEVRITKGTMVMLPLYNINRSKFIYGEDAEEFRPERWLDDATDSLNSIEAPYGHQATFIS
CrtS_PYCC 6858     PVVGRDGSIINEVRVTKGTMVMLPLYNINRSKYIYGEDAEEFRPERWLEDTTESFNSIEAPYGHQATFIS
Clustal Consensus  **:*****:*****:**********:******:***************:*.*:*:***********:***

                          500       510       520       530       540       550
                   ....|....|....|....|....|....|....|....|....|....|....|....|....|..
CrtS_CBS 7918      GPRACFGWRFAVAEMKAFLFVTLRRVQFEPIISHPEYEHITLIISRPRIVGREKEGYQMRLQVKPVE
CrtS_PYCC 6859     GPRACFGWRFAVAEMKAFLFVTLRRVQFEPIISHPEYEHITLIISRPRIVGREKEGYQMRLQVKPVE
CrtS_PYCC 6858     GPRACFGWRFAVAEMKAFLFVTLRRVEFEPIISRPEYEHITLIISRPRIVGREKEGYQMRLKVKPVG
Clustal Consensus  **************************:******:***************************:****
```

Figure S2. Amino acid alignments of the proteins involved in the astaxanthin biosynthetic route in *Phaffia* spp.

```
                               10        20        30        40        50        60        70        80        90       100
                       ....|....|....|....|....|....|....|....|....|....|....|....|....|....|....|....|....|....|....|....|
P. australis_Ste3-1    MNDAIFPVFAAIALLLLVLSCYPHFRTGNIGAIALVLWCFTSNLLYFIDCLVYWNTTNNLTPVWCDIMVKIQATTQTGLAAACLCINRRLAIISCSKQTS
                              110       120       130       140       150       160       170       180       190       200
                       ....|....|....|....|....|....|....|....|....|....|....|....|....|....|....|....|....|....|....|....|
P. australis_Ste3-1    ATSKSRRWAFWSDMLICALAPIIVAIISYCVQSHRYNIVENFGCSGSPWMDVYAILGSYGPPMLLTIVSSIYGAVAIYNFIVQRRRFQAVLQQNSSLNTC
                              210       220       230       240       250       260       270       280       290       300
                       ....|....|....|....|....|....|....|....|....|....|....|....|....|....|....|....|....|....|....|....|
P. australis_Ste3-1    RFVRLIGVAGVNIVISLLFAIRETVIAARSVYSTVSWDYIHYDFDLILTFNSAFLLGEPQLWIELNLCRWLPCLASFIYFAFFGMHEDVLSYYTYIWARS
                              310       320       330       340       350       360       370
                       ....|....|....|....|....|....|....|....|....|....|....|....|....|....|....|..
P. australis_Ste3-1    SQALLHAKERIFGRPPPVYDPSQYPKLGTAVASPSERGWSRNGMPDEEQAFPYSHMETSEKRDTSEHPLPRIEKVVS
```

```
                               10        20        30        40        50        60        70        80        90       100
                       ....|....|....|....|....|....|....|....|....|....|....|....|....|....|....|....|....|....|....|....|
P. australis_Ste3-2    MRDALFPIYSGLAIILLLIACGPHIRAGNVGAISLMTWCMLANVTYFINALIYQDTAADLTPIWCDIVVKIQCAVQTGLAASCLCINRRLATISSPTQSR
                              110       120       130       140       150       160       170       180       190       200
                       ....|....|....|....|....|....|....|....|....|....|....|....|....|....|....|....|....|....|....|....|
P. australis_Ste3-2    QTETSRQWAFWSDVAICLVPTALVLIVSYCVQAHRYNIIENFGCFPATWLELYAILGLFVPPVLCAAGSFICGGFAIYNFLAQRRRFQAVLQQNSSSLNS
                              210       220       230       240       250       260       270       280       290       300
                       ....|....|....|....|....|....|....|....|....|....|....|....|....|....|....|....|....|....|....|....|
P. australis_Ste3-2    SRLRLVGVAAVDMVLSLPFGVYEIIHNAYTLQPTYSWADLHDNFNLVKEIDQSILNAQPGSWASINLSRWMTTLAAFVYFAFFGMHEDALSFHASIWTKI
                              310       320       330       340       350       360       370       380
                       ....|....|....|....|....|....|....|....|....|....|....|....|....|....|....|....|....
P. australis_Ste3-2    SAGFGYIWIRFFGKSEPPSYDSSQLTQLGTTIASPSDQNRFCNSIKDEEAYFAYENMTGLEKSDSSLGVDSKIFVTVNIQRTIS
```

```
                               10        20        30        40        50        60        70        80        90       100
                       ....|....|....|....|....|....|....|....|....|....|....|....|....|....|....|....|....|....|....|....|
P. tasmanica_Ste3-1    MSDAVFPVFASLALLLLILSCYPHFRTGNIGAITLVIWCFLSNFFYFVDCIVYWDTVKNLTPVWCDIMVKIQATTQTGLAAACLCINRRLAIISCSTQTN
                              110       120       130       140       150       160       170       180       190       200
                       ....|....|....|....|....|....|....|....|....|....|....|....|....|....|....|....|....|....|....|....|
P. tasmanica_Ste3-1    ITSKARRRAFWSDMLICALAPVVVAVVSYCIQSHRYNVIENYGCAGVPWLDTYAIIGLYGPSLLMGFISSIYGAVAIYKFVAQRRRFQMVLQQNSSLNTS
                              210       220       230       240       250       260       270       280       290       300
                       ....|....|....|....|....|....|....|....|....|....|....|....|....|....|....|....|....|....|....|....|
P. tasmanica_Ste3-1    RFVRLIGVAGINIVISLLFAIRETVLATRFRDPTVSWDYIHDDFSLVIPIDSSYLSENPQLWVELNLSRWLPCLASYIYFAFFGMHEDVLAYYTYVWMRS
                              310       320       330       340       350       360       370       380
                       ....|....|....|....|....|....|....|....|....|....|....|....|....|....|....|....|...
P. tasmanica_Ste3-1    SEALSRTRERLFGRASPVYDPSQYPKLGTTVASPSERVWSRERNPDEEEDLPYSHMEISEKNDTEDRSSPHVTVTIEVEKVVS
```

```
                               10        20        30        40        50        60        70        80        90       100
                       ....|....|....|....|....|....|....|....|....|....|....|....|....|....|....|....|....|....|....|....|
P. tasmanica_Ste3-2    MKDVLFPVYSGFAVILLLMACGPHVRAGNVGAISLMTWCMLANITYFINALIYQDTAENLTPIWCDIVVKIQCAVQTGLAASCLCINRRLATISSPTRSR
                              110       120       130       140       150       160       170       180       190       200
                       ....|....|....|....|....|....|....|....|....|....|....|....|....|....|....|....|....|....|....|....|
P. tasmanica_Ste3-2    QTETSRQWAFWSDIAICLVPTTLVLIVSYCVQAHRYNIIENFGCFPATWLELYAILGLYIPPVLCAACSFGFGGFAIYNFLAQRRRFQAVLQQNSSALNS
                              210       220       230       240       250       260       270       280       290       300
                       ....|....|....|....|....|....|....|....|....|....|....|....|....|....|....|....|....|....|....|....|
P. tasmanica_Ste3-2    SRFLRLIGVAAVDMVLSLPFGIYEIIHNTRNLQPTYSWADIHENFGLVKEIDLSVLNESDSWTSVDLSRWMTTLAAFIYFAFFGMHEDALLFYASIWTKI
                              310       320       330       340       350       360       370       380
                       ....|....|....|....|....|....|....|....|....|....|....|....|....|....|....|....|....
P. tasmanica_Ste3-2    SAIFSYPWMRTSKMSEPPSYDSSQLTQLGTTVASASDLERFCNTIKDEEAYFAYENMIGSEKSGSDMGVDSKIVVTVKIEKTIS
```

Figure S3. Predicted proteins of the pheromone receptor genes of *P. australis* and *P. tasmanica*. The transmembrane domains of each sequence are highlighted in blue.

```
                  10        20        30        40        50        60        70        80        90       100
P. australis_Hd1  MESFPASDLSAPLDSLVDKFLNSIRAGIPVEISQIYDAFAEERRKARNEGLLTFEYQEEISASVGFLSTLASCMMNIPEDKTDFQTSVIGILNSANNIPE
                 110       120       130       140       150       160       170       180       190       200
P. australis_Hd1  VCPPRFLPSPLNTLPPKPDSERSMRPRTEHPSSFRHLRDWFIVHLDHPYPSAQEKEELALAKNLTKNSVNLWFNNMRRRSGWMDFVRTHALNNQNRMKEL
                 210       220       230       240       250       260       270       280       290       300
P. australis_Hd1  VLLHRSLPAVEQSHPELPFELRQQLDRIQDHVDEKGHDKVRDWMAGALDYRESSGVTVEEIGRFPDKNKEVENDTTKTDSSSDLYKQASSATKEAQHWLD
                 310       320       330       340       350       360       370       380       390       400
P. australis_Hd1  PYLTSTSGLVGYLPSVPLSNRPTQKSSKRSFSDHVPHVAASLDPVSNYHQLINFPNRPDQNREGGMQTHQPVPTHAYYPTYPQLQKPLPLLGPIPYQSSF
                 410       420       430       440       450       460       470       480       490       500
P. australis_Hd1  NSQFPAYDLNTSNRAQAWLPPHPVVDLSGGPNIHQRSVSPLPSRAKSTGPSLSITTTPQSIRSSANESAGNELAAKLEALRIKKEALKKEKEQLRAEAEA

P. australis_Hd1  LGEVFDC

                  10        20        30        40        50        60        70        80        90       100
P. australis_Hd2  MNSLSKIPQMSVGLGDVFDSDLDLLTSKEFTIEDLYLPVPTSICETLEELGTPHTLARNLGALYLSRAFALRELSLLQFDTAFPADRTFLLDANSLKLKV
                 110       120       130       140       150       160       170       180       190       200
P. australis_Hd2  FKGLLKATLEHRYSVELSHHQETIIQLAGVWTSDDEQTPVSVRRSSFTKNTIDTLQALYDLNQYPNPVELKTIAVKVGLQPKQIRAWFQNRRNRRLSPAQ
                 210       220       230       240       250       260       270       280       290       300
P. australis_Hd2  SQKKTPMSLKRKIAASDLNLNTVHKRRSRGTASFENEQEDQSVPSRLGSPISLQTEKASIVPDTQGRLKACCPSVADLFDTPSNDQDASTPWSTIISDGP
                 310       320       330       340       350       360       370       380       390       400
P. australis_Hd2  SIYASFEQVPQIESSLPFAIPEPADLMSVQIADGSSNVSNKSFSSHPVYTPALNLNETTKTHPVDWRMVFAKSNSRSLAPRFEEVGAQLNSKMRRGIKNV
                 410
P. australis_Hd2  IIDVSPITTTER

                  10        20        30        40        50        60        70        80        90       100
P. tasmanica_Hd1  MESYPTSDLSARLDSLVEKFLNPIRAGVPVDLSQIYDAFAEERRKAWSEGLLTFEFQEEISASVGFLSTLASCMTNTPDENTDFQTTVIGILNDANNASE
                 110       120       130       140       150       160       170       180       190       200
P. tasmanica_Hd1  ICPPRHLTLPSNTLPLKADTGRVMRPRTEHPSSFRHLRDWFIVHLDHPYPSAQEKEELALAKNLTKNSVNLWFNNMRRRSGWMDFVRTHAVNNQNRMREL
                 210       220       230       240       250       260       270       280       290       300
P. tasmanica_Hd1  VLLHRSLPASERSHPELSFELRQQLDRIQDHVDEKGHDRVRDWMTGALEYKDSFEGTADRTDQSLEKRKEKGKGTTRSDFSSDLYKKSFTTKDVHPWINP
                 310       320       330       340       350       360       370       380       390       400
P. tasmanica_Hd1  YPAPASGLIGYPPSVPLVNRPVQNSSKRSFSEDDSYVAAPIDAVSDYHQLMSLSNGPNQQHEGSLHTHQPLPAYAYHPTYPQLRNPLPMLGPTSYHSSSD
                 410       420       430       440       450       460       470       480       490       500
P. tasmanica_Hd1  PQFPAYDLNASTRAQTWLSSNPAGVLSGGPKVHQRSVSPLPSRAKPVLSSLASSTTTPSSRSSANESAGNELEAKLEALRIKKEALKKEKEQLRVEAEAL

P. tasmanica_Hd1  GEVFDC

                  10        20        30        40        50        60        70        80        90       100
P. tasmanica_Hd2  MNSLPKFPQKSTGPGDVFDSDRGAPSSSEFATEDLDLPVPMSICETLEELGTPRALARNLGALYLSKASALRELSLMQFDAAFPAERSFLLETNSLKLKV
                 110       120       130       140       150       160       170       180       190       200
P. tasmanica_Hd2  FKGLLKATLEHRYSMELTHYQELIIQLAGVWTSDDEQPSVTVRRSSFTKNTIDTLQALYDLNQYPNPVELKTIAAKVGLQPKQIRAWFQNRRNRRTSPAR
                 210       220       230       240       250       260       270       280       290       300
P. tasmanica_Hd2  PHKKALKTSTRATAASHLKIDIVRKRSPSRTISIETETKDEGVPSRLGSPISFQTNRISSVPQPQERPRDGHSSVANFFETSPKKQDSSALWLAVSSDRS
                 310       320       330       340       350       360       370       380
P. tasmanica_Hd2  STYPNFEGPPQIGSSLHFSTPEPADLMNVRLAEDPFDLRNKSTSSHPVFTPVFGSNEIMDIDPMNWRMIFATPNSRTVAPGSRKVGAQ
```

Key: Homeodomain Coiled-coil Nuclear localization signal Alpha-helix

Figure S4. Predicted proteins of the homeodomain transcription factor genes of *P. australis* and *P. tasmanica*. The secondary organization is highlighted.
